# Dementia risk estimation in persons at risk and the predictive turn in Alzheimer’s disease—The PreTAD project: Study protocol with an ethical, clinical, linguistic, and legal approach

**DOI:** 10.1371/journal.pone.0319868

**Published:** 2025-07-16

**Authors:** Michelle Gerards, Annika Baumeister, Constanze Hübner, Federica Ribaldi, Yahveth Cantero-Fortiz, Julia Braun, Mercè Boada, Giovanni B. Frisoni, Frank Jessen, Björn Schmitz-Luhn, Carolin Schwegler, Christiane Woopen, Ayda Rostamzadeh

**Affiliations:** 1 Department of Psychiatry and Psychotherapy, Medical Faculty, University of Cologne, Cologne, Germany; 2 Center for Life Ethics, TRA 4, University of Bonn, Bonn, Germany; 3 Geneva Memory Center, Department of Rehabilitation and Geriatrics, Geneva University Hospitals, Geneva, Switzerland; 4 Laboratory of Neuroimaging of Aging (LANVIE), University of Geneva, Geneva, Switzerland; 5 Ace Alzheimer Center Barcelona—Universitat Internacional de Catalunya, Barcelona, Spain; 6 Networking Research Center on Neurodegenerative Diseases (CIBERNED), Instituto de Salud Carlos III, Madrid, Spain; 7 German Center for Neurodegenerative Diseases (DZNE), Bonn, Germany; 8 Excellence Cluster on Cellular Stress Responses in Aging-Associated Diseases (CECAD), University of Cologne, Cologne, Germany; 9 Faculty of Arts and Humanities, University of Cologne, Cologne, Germany; PLoS ONE, UNITED STATES OF AMERICA

## Abstract

**Background:**

Despite progress in the field of Alzheimer’s disease (AD) dementia risk estimation, little is known about its impact at the individual and societal levels.

**Objective:**

Introducing the explorative tri-national PreTAD project (The Predictive Turn in Alzheimer’s Disease: Ethical, Clinical, Linguistic and Legal Aspects), which aims to (1) learn about attitudes, needs, and perspectives on AD dementia risk estimation of the general population and cognitively unimpaired individuals with and without contact to memory clinics, (2) identify anticipated impacts of AD dementia risk estimation and (3) discuss the implications of the paradigm shift in medicine at individual and societal levels from an ethical, linguistic and legal perspective.

**Methods:**

Different approaches are used: (1) an assessment of a population without experience with dementia, (2) an assessment in memory clinics, and (3) an online survey of the general population. Participants include cognitively healthy adults (n=2760), first-degree relatives of dementia patients (n=150), and participants with existing (n=150) and newly diagnosed (n=90) subjective cognitive decline (SCD) from Germany, Switzerland, and Spain.

**Results:**

As part of the PreTAD project, new questionnaires are developed that (1) collect attitudes, needs, and perspectives on AD dementia risk estimation and (2) assess anticipated impacts of dementia risk estimation using hypothetical blood-based biomarker dementia risk scenarios.

**Conclusion:**

The PreTAD study combines an interdisciplinary approach to develop a framework for predictive medicine in the preclinical stages of AD and supports improving communication of biomarker-based dementia risk estimation in clinical practice. The study was registered in the German Clinical Trials Register (DRKS00029035 on 03/08/2023).

**Trial registration:**

German clinical trials register (Deutsches Register Klinischer Studien, DRKS): http://www.drks.de/DRKS00029035, DRKS registration number: DRKS00029035, date of registration: 08.03.2023.

Protocol version 3.0, date 01.06.2024

## Introduction

According to the International Working Group (IWG) 2024 criteria, Alzheimer’s disease (AD) diagnosis includes individuals with positive biomarkers and specific Alzheimer’s phenotypes, while biomarker-positive, cognitively unimpaired individuals are considered at risk for AD [1]. Due to the emotional and economic burden associated with AD, the hope for effective treatment is rising. With emerging blood-based biomarkers, testing will be facilitated and may be offered not only in prodromal but also in preclinical stages. Therefore, the desire and request to perform biomarker testing for AD dementia risk estimation from cognitively unimpaired individuals and those with subjective cognitive decline (SCD) may increase. As the IWG 2024 criteria distinguish individuals with AD from individuals at risk for clinical progression to prodromal AD or AD dementia based on the Alzheimer’s phenotype, recommendations for biomarker assessment for cognitively unimpaired individuals and individuals with SCD might be very different.

There are arguments for dementia risk estimation using biomarker testing in prodromal and preclinical stages. Addressing modifiable lifestyle-associated risk factors could prevent up to 45% of dementia cases [2]. Initiatives like the Brain Health Services for dementia prevention (BHS) propose four cornerstone interventions: 1) risk assessment, 2) risk communication, 3) risk reduction, and 4) cognitive enhancement [3]. Furthermore, ongoing clinical trials focus on disease-modifying drugs in preclinical stages [4], hinting at future pharmacological interventions for disease prevention at early stages.

SCD with positive biomarkers may be defined as a preclinical stage of AD, in which subjective experience of cognitive decline is noted, whilst performance on neuropsychological tests is within normal limits and activities of daily living are maintained [5]. While SCD refers to a non-specific and heterogeneous syndrome, recent research showed an association between SCD and AD biomarker evidence with an increased risk for cognitive decline and autoptic hallmarks of AD [6–8]. With technological advances, new sensitive, specific, and reliable markers will be developed for identifying individuals at risk for cognitive decline, for differential diagnosis of dementia, and the monitoring of disease progression [9]. However, to date, biomarker testing is not recommended for asymptomatic individuals in clinical routine, due to insufficient predictive accuracy and the lack of approved disease-modifying medication for asymptomatic individuals [10,11]. However, early disease risk estimation may also be a chance for action-taking, such as lifestyle modification to reduce the modifiable dementia risk factors [2,12]. A further challenge is the communication of disease probabilities, which may result in patient uncertainties due to an unclear conceptual understanding of predictive diagnostics and risk probabilities, distinct from definitive diagnoses [13]. In particular, when communicating screening results, there is a possibility of confusing a disease risk with a disease diagnosis [14].

Recommendations and guidance on counseling and disclosure of biomarker test results for cognitively healthy individuals and SCD patients in the clinical setting are sparse, particularly regarding blood-based biomarker testing [15,16]. Shaw et al. developed appropriate use criteria (AUC) for CSF AD biomarker testing. According to the AUC, the use of CSF biomarker testing of asymptomatic individuals without a high risk or SCD symptoms, as well as SCD patients who are not considered at increased risk for AD, was classified as inappropriate. In contrast, biomarker testing of individuals at increased risk for cognitive decline was classified as appropriate. The workgroup suggests testing in this population only when it aligns with patient goals and after a full discussion of potential biomarker test outcomes [17]. According to the AUC for amyloid PET, amyloid imaging was classified as inappropriate for patients with unconfirmed cognitive complaints, solely based on positive family history or APOE ε4, and for asymptomatic individuals [15]. Limited data on the psychological outcomes after AD biomarker test result disclosure in cognitively normal research participants revealed no major psychological harm [18,19]. It is recommended to provide information sheets on AD biomarker results with educational content and additional measures for screening of anxiety and depression symptoms, assessments of mood and readiness to receive results, a written report, and telephone follow-ups to assess the impact of disclosure [16,18]. Ketchum and colleagues [16] have introduced a conceptual model with clinically relevant phases of care in preclinical AD. Using the framework of Huntington’s disease, this model distinguishes pre‐disclosure, disclosure, and post‐disclosure stages for AD biomarker testing. The authors highlight important areas for further research before implementing preclinical biomarker testing in routine clinical care.

The predictive turn in AD touches upon fundamental ethical values and concepts, including questions of autonomy, physical, mental, and social health and well-being, justice, and solidarity. For example, biomarker-based AD diagnosis in preclinical populations raises important ethical and legal issues regarding the process of informed consent and disclosure of test results [20,21], especially when considering that the knowledge of a high risk of developing dementia potentially affects all areas of an individual’s life and social environment. Additionally, questions on insurance and social law [22], especially concerning statutory health care, arise. As practical standards for counseling on predictive AD diagnostics are still not well developed, ethical and legal aspects, such as (barriers) to shared decision-making, the right to know and the right not to know, as well as the right to withdraw consent, should be taken into account sufficiently [23]. Amongst others, two crucial aspects need to be addressed from an ethical perspective: (1) disclosure of a biomarker result that only refers to a risk and is not a definitive diagnosis, and (2) disclosure of the dementia risk in the absence of a treatment option [24]. In the process of biomarker disclosure, ethical discussions aim to strike a balance between patient autonomy and the “value of knowing” and the principle of non-maleficence [25,26]. Individuals need to be given all the necessary information to make an informed decision and have the opportunity to change their minds [20]. The issues of patient autonomy, the right to know/not know, and the weighing of potential risks and benefits are important in deciding whether and to what extent an individual wishes to undergo diagnostics [27]. It is crucial to open the discussion on the potential benefits of predictive testing (e.g., better future life planning, increased diagnostic certainty, lifestyle modification, access to clinical trials) and weigh them against the risk of harm (principle of non-maleficence with unknown psychological impact and predictive value) [24]. Ethical frameworks for counseling people in the early stages of AD must fulfill the existing law and consider individuals’ expressed wishes, such as wanting to be informed, being able to make self-determined decisions, and knowing about their right to know [27]. In addition, such frameworks should contain recommendations for clinical practice on how to strike a harmonious balance between conflicting ethical values in each case. Ethically relevant aspects and values can be affected in two ways, namely (1) by the decision-making process for or against AD dementia risk estimation and (2) by the impact that individual AD dementia risk knowledge may have [28], both for the affected individual and for society. In this regard, individual decision factors and preferences, anticipated consequences [28], as well as perceptions and concepts of health, risk, and disease, should be considered [29,30]. However, to date, empirical data are insufficient to prove the potential psychological and social benefits and risks of AD biomarker disclosure in early stages of AD [24].

As predictive methods will likely become more accessible, it is important to understand clinically, ethically, and legally relevant aspects from both an individual and a societal level [28]. The study “Predictive Turn in Alzheimer’s Disease” (PreTAD) is an explorative study following a multi-disciplinary approach to (1) learn about attitudes, needs, and perspectives on AD dementia risk estimation of the general population and cognitively unimpaired individuals with and without previous contact with memory clinics, (2) identify anticipated impacts of AD dementia risk estimation, and (3) discuss the implications of the paradigm shift in medicine at the individual and societal levels from an ethical, linguistic, and legal perspective. To achieve those goals, different approaches are used: (1) an assessment of a population without experience with dementia, (2) an assessment in memory clinics, and (3) an online survey of the general population. Participants include cognitively healthy adults (n=2760), first-degree relatives of dementia patients (n=150), and participants with existing (n=150) and newly diagnosed (n=90) subjective cognitive decline (SCD) from Germany, Switzerland, and Spain.

By examining the impact of predictive medicine on the individual, the respective society, and the health care system using the example of AD, a comprehensive framework for clinical practice will ultimately be developed.

## Methods

### Clinical and ethical approach

**Fig 1:**
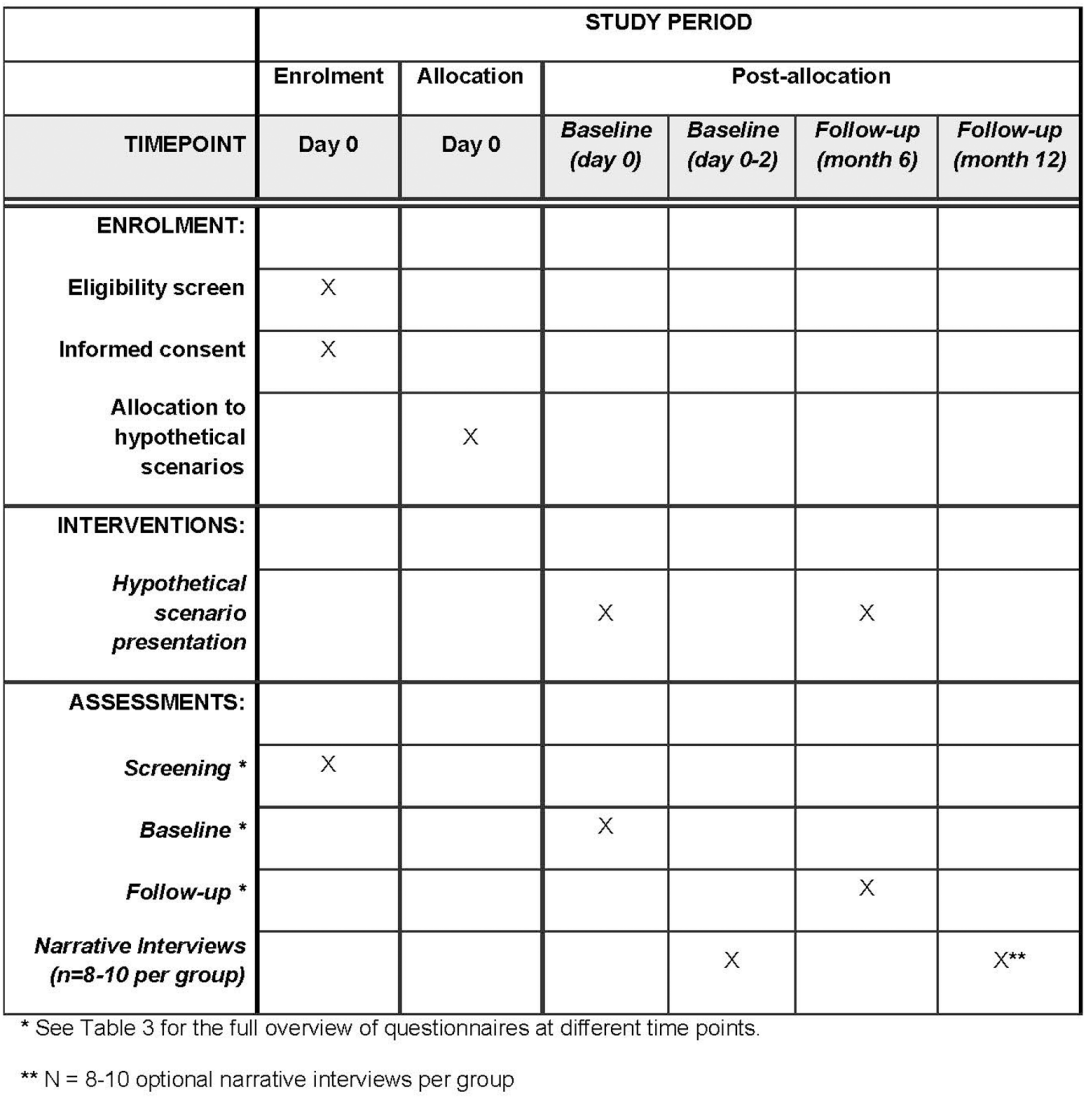
Schedule of enrolment, interventions, and assessments of the PreTAD study.

As part of the clinical and ethical approach, attention will be focused on groups that will be increasingly important for the future of AD dementia risk estimation. These include cognitively healthy individuals who may not yet have experience with AD dementia or cognitive impairment, as well as individuals affected by SCD and the general population. Therefore, we follow different approaches: (1) an assessment of individuals with SCD and first-degree relatives of patients with dementia in memory clinics, (2) an assessment of individuals from the general population without previous experience with dementia, and (3) an assessment of the general population via an online survey. Through the use of quantitative and qualitative methods, comprehensive empirical findings are to be used for an in-depth and differentiated analysis of ethical aspects that are particularly relevant to these groups. The online survey of the general population will provide insights into attitudes and expectations regarding AD dementia risk estimation, which gives additional information for the development of adequate information material and clinical guidelines. An overview of the schedule of enrolment, interventions, and assessments of the PreTAD study is shown in Fig 1.

The empirical findings are extended by an ethical discussion on a) ethically relevant aspects of AD dementia risk estimation at both individual and societal levels and b) the contextualizing of these aspects in the organization of healthcare and clinical processes to provide ethical guidance. In terms of a comprehensive yet pragmatic ethical approach, the derived ethical considerations will be embedded in all steps of the clinical process in AD dementia risk estimation, from the provision of general information about the decision-making process to the disclosure of risk and follow-up care.

**Fig 2:**
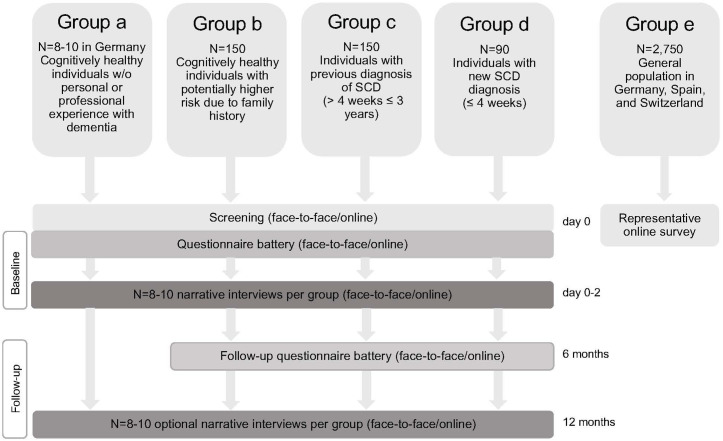
Study flow-chart. AD, Alzheimer’s disease; SCD, subjective cognitive decline.

An overview of the study plan is shown in Fig 2. Table 1 shows the data collection at the individual study sites. Inclusion and exclusion criteria are shown in Table 2. The data collection, as a combined clinical and ethical approach, is divided into a quantitative and a qualitative part. 

**Table 1:** Oversight of study sites and methods.

Study site		Method of empirical data collection
Germany (Ethics, Clinics, Linguistics, Law)	Center for Life Ethics, Bonn	Qualitative, in-person (or online) Quantitative, online, or in-person (via online tool)
	Center for Memory Disorders of the University Hospital of Cologne, Cologne	Quantitative, online, or in-person (via online tool)
Switzerland (Clinics)	Geneva Memory Center, Geneva	Quantitative; online or in-person (via online tool)
Spain (Clinics)	ACE Alzheimer Center Barcelona, Barcelona	Quantitative, online, or in-person (via online tool)

**Table 2:** Inclusion and Exclusion criteria of groups a-d for the PreTAD study.

**General Inclusion Criteria groups a-d (survey and interviews)**	At least 18 years old Sufficient proficiency in the national language of each study site so that informed consent can be given and the completion of the questionnaires can be conducted Interviews only: sufficient proficiency of the German language so that the qualitative interviews can be conducted in German
**General Exclusion Criteria groups a-d (survey and interviews)**	Insufficient knowledge of the national language Illiteracy Persons who are not capable of giving consent or who have not given written consent
**Additional Inclusion/Exclusion Criteria** **Group a**	None
**Additional Inclusion Criteria** **Group b**	Moderate to severe depressive disorder; Hospital Anxiety and Depression Scale (HADS) score > 10 points Knowledge of a medical diagnosis of Alzheimer‘s disease in at least one first-degree relative (mother, father, sibling)
**Additional Exclusion Criteria** **Group b**	Moderate to severe depressive disorder; Hospital Anxiety and Depression Scale (HADS) score > 10 points Acute suicidality
**Additional Inclusion Criteria** **Group c/d**	Clinical criteria for the diagnosis of SCD (according to the criteria of Jessen et al. 2014) > 4 weeks and ≤ 3 years (group c) or ≤ 4 weeks (group d) A subjective and persistent (non-acute) deterioration in cognitive performance compared to the original baseline level, unrelated to an acute event Neuropsychological test battery used for mild cognitive impairment (MCI) or prodromal AD indicates a finding within the age-, gender-, and education-adjusted norm group
**Additional Exclusion criteria** **Group c/d**	MCI, prodromal AD or dementia Impairments can be explained by a psychiatric* or neurological illness (excluding AD), somatic illness, medication, or substance abuse Moderate to severe depressive disorder; HADS score > 10 points Acute suicidality

*Mild subsyndromal depressive symptoms or anxiety symptoms are not considered as an exclusion criteria.

#### Quantitative study.

The questionnaire battery includes validated questionnaires used in memory clinics and newly developed questionnaires. Assessments take place at baseline and follow-up after 6 months (Table 3). Newly developed questionnaires assess previous experience with dementia (see *Previous experience with dementia*), attitudes, needs, and perspectives on AD dementia risk estimation (see *Needs and decision parameters*) and anticipated impacts of AD dementia risk estimation based on hypothetical risk scenarios of AD dementia (see *Hypothetical scenarios of plasma biomarker testing*). Within the hypothetical scenarios, participants are confronted with one of two hypothetical risk scenarios:

**Table 3:** Overview of questionnaires. BRS, Brief resilience scale; FLZ^M^, Questions on life satisfaction modules; HADS, Hospital anxiety and depression scale; HS1, Hypothetical scenario - increased AD dementia risk; HS2, Hypothetical scenario – standard AD dementia risk; MMSE, Mini-mental state examination; SCDQ, Subjective cognitive decline questionnaire; UCLA, University of California Los Angeles.

Questionnaires	Groups a-d				Group e
**Screening:**					
MMSE [31]	X				No screening
HADS [32]	X				
SCDQ [33]	X				
	**Baseline**		**Follow-up**		**One-time survey**
	**HS1**	**HS2**	**HS1**	**HS2**	
HADS [32]			X	X	
Demographics	X	X			X
Previous experience with dementia	X	X			Abbreviated
Needs and decision parameters	X	X	X	X	Abbreviated
Subjective risk perception [34]	X	X	X	X	X
Lifestyle [34]	X	X	X	X	
FLZ^M^ [35]	X	X	X	X	
Hypothetical scenario increased risk	X		X		Abbreviated
Hypothetical scenario standard risk		X		X	
Subjective numeracy [36]	X	X			
Objective numeracy [37]	X	X			X
UCLA loneliness scale (3-items) [38]	X	X	X	X	
BRS [39]	X	X	X	X	X
Big five (10-items) [40]	X	X			X

Study arm 1) High risk of developing AD dementia due to abnormal biomarker results or
Study arm 2) A lifetime dementia risk comparable to the general population

The participants of groups a-d are randomized in a 1:1 allocation to each study arm, receiving either the hypothetical scenario 1) (HS1) or the hypothetical scenario 2) (HS2).

To ensure that participants are not cognitively impaired, the Mini-Mental State Examination is used for screening [31]. Furthermore, a questionnaire to assess SCD based on the criteria proposed by Jessen et al. is conducted to ensure that participants are assigned to the correct groups [33].

Many individuals lack the basic numerical skills required to navigate the modern healthcare system effectively [41]. Statistical numeracy is crucial for autonomous and informed decision-making in predictive medicine, where risks are typically communicated in probabilities [42]. Studies have shown that limitations in health numeracy reduce the accessibility of risk and decrease the benefits of risk estimation [43]. Furthermore, people with lower numeracy skills tend to be more susceptible to bias when health-related numerical information is presented in a particular manner [44], leading to higher demands on physicians’ risk communication abilities [43]. To assess difficulties in dealing with complex risk estimation, questionnaires on subjective and objective numeracy are used [36,37]. To measure subjective risk perception, a questionnaire developed in the PreDADQoL project, assessing the subjective appraisal of the individual risk for developing AD dementia, is applied [34]. An additional item asking about the participant’s perception of what is perceived as an increased risk of AD dementia was added.

To gain insights into the factors of lifestyle and life satisfaction, the lifestyle questionnaire developed in PreDADQoL [34], and the validated questionnaire on satisfaction with life (FLZ^M^) [35] are applied.

One concern of early AD diagnosis and AD dementia risk estimation is the psychological impact of dementia risk disclosure. Especially due to the chronic neurodegenerative character of the disease and the absence of curative treatment options, individuals are facing severe impacts on their lives. To assess psychological and social distress, the Hospital Anxiety and Depression Scale (HADS) [32] and the three-item UCLA Loneliness Scale [38] are used.

Research has shown that personality traits affect preferences in medical decision-making [45]. To assess personality traits that are potentially linked to certain preferences and needs regarding AD dementia risk estimation, as well as the impact of personality and resilience on dealing with risk estimation, an abbreviated ten-item version of the Big-Five questionnaire [40], and the Brief Resilience Scale (BRS) are applied [39].

#### Qualitative study.

For the qualitative part, narrative interviews are conducted to reveal aspects of interest. The interviews are conducted at the site of Cologne only. Eight to ten participants per group (a-d) are included in the interview study to gain in-depth insights into individual attitudes, preferences, and needs regarding risk estimation and to examine how characteristics may affect the decision for or against dementia risk estimation.

The interview structure is intentionally loosely specified, following the intended nature of a narrative interview [46]. This approach was chosen to create a natural flow of conversation between the researcher and participant. The interview is divided into three thematic sections that serve as reference points for the researcher, and to stick to the objective in case the participants digress from the actual question. The three thematic sections are organized as follows (types of questions are presented exemplarily):

[1] Participants’ individual (previous) experiences and attitudes regarding AD dementia and AD dementia risk estimation: participants are asked about their experience with dementia and their views on risk estimation measures in general and AD dementia risk estimation in particular.
[2] Points of access and needs: Among others, questions are asked about access to the memory clinic (applicable to groups c-d) or needs and wishes regarding AD dementia risk communication and psychosocial support (applicable to groups a-d).
[3] Contextualization and associations: In the last part of the interview, participants are asked to reflect on thoughts and emotions they associate with terms such as ‘health’, ‘disease’, and ‘risk’. Since this can be considered the most explorative part of the interview, participants can also draw their associations or emotions into ‘body maps’ [47]. Body maps are a technique mostly applied in qualitative research. They can help to understand individual embodiment (e.g., feeling of ‘risk’). This technique can support participants who might have problems expressing their feelings and attitudes verbally or are not willing to do so [48]. The application of body maps in the context of SCD provides insightful information about risk-related perceptions or visions of one’s state of health [49].

#### Memory clinics.

PreTAD addresses three groups of participants with contact to memory clinics to consider the different views of cognitively unimpaired people, who had previous direct or indirect contact with memory clinics. The participants are divided into group b) people with potentially higher risk due to family history (n=50 participants per site, n=150 total), group c) people with previously diagnosed SCD (n=50 participants per site, n=150 total), and group d) people with newly diagnosed SCD within the last 4 weeks before screening (n=30 participants per site, n=90 total) (Fig 2). General inclusion criteria contain a minimal age of 18 years, and sufficient proficiency in the national language. Additional inclusion criteria for group b include the knowledge of a medical diagnosis of AD in at least one first-degree relative. Additional inclusion criteria for groups c and d embrace the clinical criteria for the diagnosis of SCD >4 weeks and ≤3 years (group c) or ≤ 4 weeks (group d) and an age-, gender- and education-adjusted normal result on neuropsychological testing . General exclusion criteria include insufficient knowledge of the national language, illiteracy, and inability to provide consent. Additional exclusion criteria for group b include a subjective or objective cognitive impairment and a moderate to severe depressive disorder (HADS >10 points). Additional exclusion criteria for groups c and d include the clinical diagnosis of MCI or dementia, possible explanations by other psychiatric or neurological illnesses, somatic illness, medication or substance abuse, and moderate to severe depressive disorder (HADS >10 points). Assessments take place as described in Table 2. The participants are recruited through Alzheimer’s prevention registries (group b) and memory clinics (groups c/d).

#### General population without previous experience with dementia.

Participants of the general population without any family history or previous personal experience with dementia are included in group a (Germany only, n=8–10 participants). They will be assessed in the same manner as participants in groups b-d as described above (Table 2). This group is recruited through social networks and public facilities.

#### General population.

The survey of the general population (group e) includes individuals from Germany (n=1000), Spain (n=1000) and Switzerland (n=750). The general population participates in a one-time online survey (Fig 2). Validated and newly developed questionnaires are used (Table 2). The questionnaire on anticipated impacts of AD dementia risk estimation consists of only one hypothetical scenario, indicating a high risk of AD dementia (based on the perception of the respective respondent of what is considered a high risk). The survey includes questionnaires used in the memory clinic assessments. It is abbreviated to serve better handling and compliance with reduced time expenditure for participants of the general population, who answer the survey online from home. The survey is distributed by the external service provider Dynata, a large first-party data platform. For more information, see https://www.dynata.com/ [50]. The quantitative data of group e are processed with the GDPR-compliant guidelines of the corresponding external service provider. For the online survey, sample sizes were calculated to achieve a reasonably representative sample of participants for each country. Quota sampling is used according to age and gender, and raw data are weighted to achieve representative samples in three countries.

***Regulatory review and approval*:** The institutional review boards of each participating institution in Germany (Business Ethics Committee, Faculty of Medicine, University of Bonn, Bonn on 14 November 2022, approval number 356/22; Office of the Ethics Committee, Faculty of Medicine, University of Cologne, Cologne on 26 June 2023, approval number 22-1335), Spain (Ethics Committee for Research of the Hospital Universitari de Bellvitge on 14 September 2023, approval number ACE20-00115), and Switzerland (Cantonal Pharmacist Service, Cantonal Commission for Research (CCER), Geneva on 23 January 2024, approval number 2023-01500) have approved the PreTAD project. Written informed consent is obtained from all study participants (groups a-e) before participation in the study. Study participants are informed about potential risks and protective measures and can withdraw from the study at any time. The data obtained is stored in pseudonymised form. All participants were informed about the local data protection guidelines. Only researchers at the participating locations have access to the pseudonymised data.

***Trial status and timeline*:** This study is registered in the German clinical trials register (Deutsches Register Klinischer Studien, DRKS): http://www.drks.de/ DRKS00029035, DRKS registration number: DRKS00029035, date of registration: 08.03.2023. Patient recruitment began on 26 June 2023 and is expected to be completed by June 2025. The follow-up visits are expected to be completed by December 2025, finalizing data collection of the study. Partial results of the study will be published as the study progresses. Results of the overall study will be published after the end of data collection, with the aim of publishing the main results by December 2026.

### Linguistic approach

The objective of the linguistic part of the project is to identify relevant individual, societal, and scientific positions, values, and knowledge-related aspects concerning AD, early AD dementia risk estimation, and predictive medicine in general, on a sociolinguistic level. The analysis focuses on communication and language to follow the evidence of language patterns that are used to introduce, negotiate, and explain respective topics in various key contexts. In doing so, these linguistic patterns reveal individual positions as well as societal values [51]. The mentioned contexts and objects of analysis are (1) the public media/newspaper discourse, built by a systematic continuous corpus constitution via press archives such as Nexis (2) the medical expert discourse, consisting of a continuous corpus of medical publications compiled via PubMed, and (3) interview communication with participants (see *qualitative study*). The linguistic approach will contribute to the project’s overall question about the meaning of predictive medicine for the human being, its needs, perspectives, and communication by providing results from the interwoven discursive and interactional dimensions of the analysis and inviting a subsequent interdisciplinary discussion.

The analysis of the public media/newspaper discourse with linguistic methods [51] addresses the common knowledge, the discursive arguments, and the public attitudes and framings which can be the sources of patients’ expectations in clinical situations [52]. Concerning the medical expert discourse, the analysis focuses on the introduction and the framing of predictive medicine and AD dementia risk estimation in central publications, e.g., the scientific negotiation of terminology, its development, significance, and evolving specificity [53]. Despite the subsequent introduction in doctor-patient interactions or equally in study interviews, manifested clinical patterns of terminology can differ from the term’s meaning in everyday language and thereby cause linguistic and ultimately ethical challenges [53]. Considering the findings of those two textual analyses, which are connected to the underlying frame and subtle composition of verbal interactions in clinical and study contexts, the approach concludes with the above-mentioned linguistic conversation analysis to reveal interwoven linguistic patterns.

### Legal approach

The paradigm shift from diagnostic medicine to predictive medicine is accompanied by a multitude of new and until now unresolved legal issues that affect both social and private insurance law as well as treatment contracts, information, and consent, as well as liability issues.

The social insurance systems of the countries participating in the project are based on the primacy of curative medical treatment and accordingly allow primarily the reimbursement of treatment costs. Unlike medical diagnostics, however, predictive examinations only provide probabilities regarding a future onset or course of disease and not a definite diagnosis. Accordingly, the reimbursability of predictive examinations and possibly subsequent preventive treatment measures isto be clarified, including what “threshold” of a disease risk justifies what preventive measures, and how this is to be determined [54].

Increasing medical predictive capabilities go along with the risk that individuals could become increasingly “transparent” and vulnerable to stigmatization and unobjective or irrational discrimination [55]. Therefore, the aim of the legal subproject is also to identify the interests worthy of protection and effective options for their protection. In particular, answers must be found regarding how a person’s right not to know can be effectively guaranteed if predictive examinations become standard medical practice. This needs to be weighed against, inter alia, the legitimate interests of affected third parties, for example, relatives, employers, or insurers, in knowing about the predictive test results. If these results require a higher level of protection, that may still need to be established by law.

Furthermore, it is necessary to examine the requirements that must be placed on the medical information provided before a predictive examination. In principle, physicians must provide information about all factors that are important for a free decision whether to undergo – also predictive – treatment [56]. The estimation of the risk for future disease, however, is much more complex than a simple intervention like the treatment of a fracture – and depends on many more non-medical factors, including the social impact, the impact on life planning, or available financial planning options. However, the law has not yet established a list of such factors for which there is a duty to inform, or where a personal responsibility to foresee far-reaching impacts as the “general risk of life” can be assumed to lie with the patient. This, in turn, also affects potential liability claims for sub-standard information.

In the end, the legal subproject will carve out these and other questions for a legally sound, ethically justified framework for the responsible use of predictive possibilities, and inform the medical community, as well as politics and lawmakers for an equitable and sustainable regulation of the unresolved issues.

## Pilot study

A pilot study with N=10 cognitively unimpaired individuals and N=10 participants with SCD was performed in Cologne between January and February 2023 to 1) pilot the first version of the newly developed questionnaires, 2) to test the feasibility of the hypothetical scenarios of plasma biomarker testing, and 3) pilot the interview guide for the qualitative interviews. The questionnaires and hypothetical scenarios were tested for feasibility and comprehensibility in a paper-pencil version and revised based on participants’ feedback. The interview guide for the qualitative study was pre-tested with N=2 cognitively unimpaired individuals and N=6 participants with SCD. The revised, abbreviated quantitative survey administered to the study group e) was piloted in April 2023 in Bonn with a total of N=24 individuals.

After the two-stage piloting process, adjustments were made regarding format, technical bugs, and wording according to the study evaluation and feedback of participants. Individual items were adjusted for comprehensibility. Participants perceived questionnaires as comprehensible and suitable for the study’s objective.

## Results of the pilot study

### Newly developed questionnaires

#### Previous experience with dementia.

A new 13-item questionnaire was developed after expert meetings and a pilot phase (see *Pilot study*). Participants are questioned about their previous experiences with dementia. They are asked to assess their knowledge on the subject, give information on potentially affected family members, and personal and professional experience with the care of people with dementia.

#### Needs and decision parameters regarding AD dementia risk estimation.

Research on attitudes and decision parameters regarding AD dementia risk estimation is lacking. In other medical fields, the decision for or against risk estimation often depends on factors like anticipated psychological stress or fear of loss of control [57]. Research indicates that in risk communication, the source of information, considering individual needs and problem areas, and understanding the potential benefits of lifestyle changes based on risk presentation are important [58]. Furthermore, not only the direct risk communication but also other (nonmedical) sources of information contribute to attitudes and the knowledge basis for decision-making, for instance, the social environment, private experiences as well as public discourse, press, and (social) media [30]. Recent analyses of the press discourse about AD dementia risk estimation in Germany describe how press headlines and typical exaggerations support overly high expectations and preconceptions [52,53]. A flexible, patient-centered approach is needed [59–61]. To better understand what a patient-centered approach could look like, a new 33-item questionnaire was developed after expert meetings and a pilot phase to assess the needs and decision parameters of different groups of healthy individuals with various experiences with AD dementia. Participants receive information on dementia, AD, AD dementia, diagnostics, treatment, and prevention of AD dementia, addressing misconceptions and knowledge deficits. The topics queried include, e.g., information sources, willingness and factors influencing willingness to assess risk, and the impact of diagnostic methods and treatment options. Systematic literature searches were conducted in three literature databases (PubMed, Web of Science, PsycInfo) on attitudes, needs, preferences, and decision parameters related to risk estimation in the AD dementia context. The search for full-text publications (“risk AND estimation AND (needs OR desires) AD dementia (dementia OR Alzheimer OR neurodegenerative)”) revealed 681 results. After the screening process, no publications were considered. The search for full-text publications based on a wider search term (“medical AND risk AND estimation AND (needs OR desires)”) revealed 587 results. After the screening process, 12 publications were considered. A separate systemic search for full-text publications on decision parameters (“decision AND (parameters or factors) AND test AND (dementia OR Alzheimer OR neurodegenerative)”) revealed 219 results. After screening, 11 publications were considered, but none sufficiently addressed the research question. Because the research landscape in this field is scarce, topics and questions were developed by the expert group of the PreTAD consortium in internal meetings and in-depth consortium workshops. Some items on individual attitudes in general were derived from the European Values Study [62], and adjusted to AD dementia risk estimation.

#### Hypothetical scenarios of plasma biomarker testing.

Dealing with the test results of AD dementia risk estimation and its anticipated impact on individuals concerned can be challenging. In this field, more research is needed [63]. Research in genetic testing shows that early neurodegenerative disease identification does not have a major impact on quality of life [64]. To assess the impact of potential plasma biomarker test results on cognitively healthy individuals, a newly developed 41-item questionnaire presenting a hypothetical risk scenario is applied. Items regarding the anticipated impact of a communicated AD dementia risk are used. The study team randomly assigns participants of groups a-d to one of the following two hypothetical risk scenarios: 1) a higher risk of developing AD dementia due to abnormal biomarker results or 2) a lifetime risk comparable to the general population. Group e is only confronted with a hypothetical scenario indicating a high AD dementia risk (based on the previously stated personal perception of what is considered a high risk by the individual respondent).

**Fig 3:**
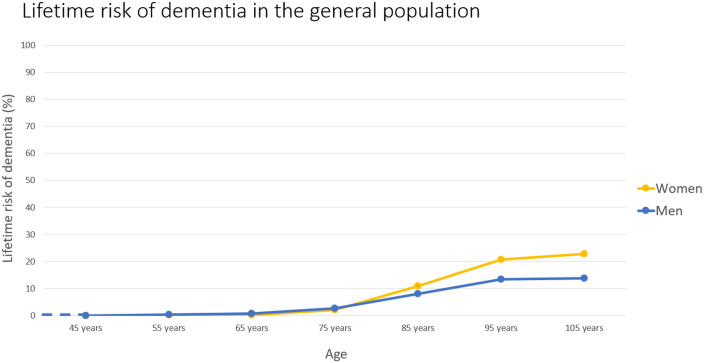
Hypothetical scenario of a lifetime risk comparable to the general population.

**Fig 4:**
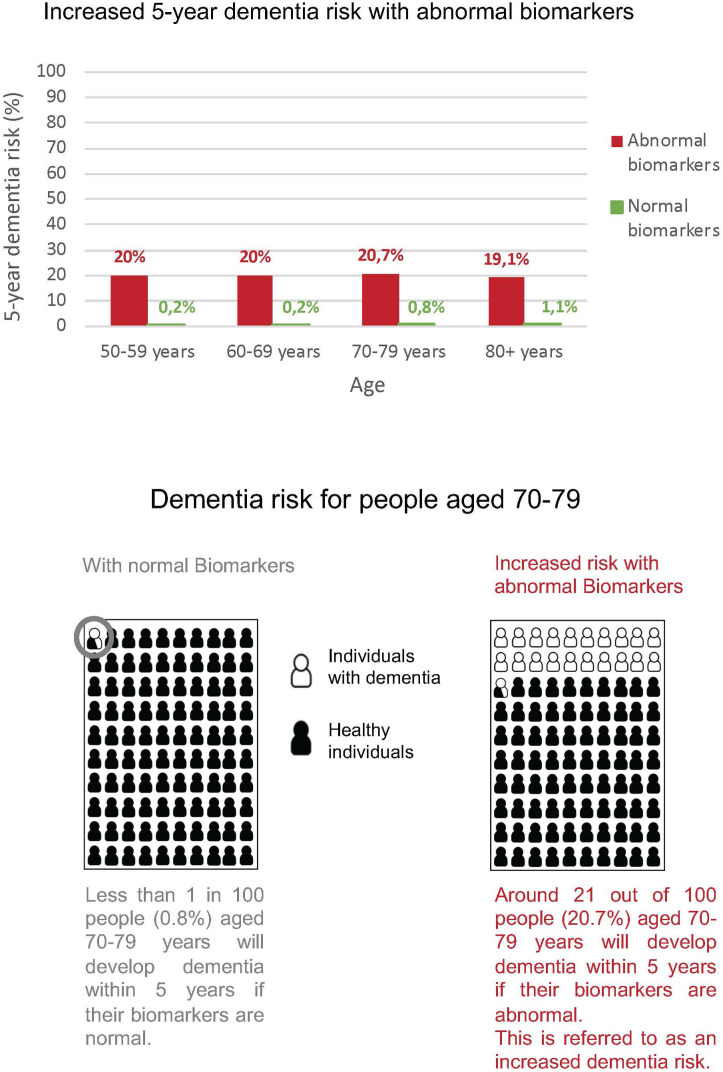
Hypothetical scenario of a higher risk of developing AD dementia due to abnormal biomarker results.

Based on a literature search, the hypothetical scenarios presented reflect the current incidence of dementia based on plasma biomarker results. Data on dementia incidence in the general population and cognitively unimpaired cohorts with known AD biomarker profiles were used [65,66]. First, a systematic search was conducted in four literature databases (PubMed, Web of Science, PsycInfo, Livivo) to identify a source for the overall dementia incidence of the general population. The search for full-text publications based on a wide search term (“incidence AND lifetime AND risk AND dementia”) revealed 297 results. After the screening process, four publications were considered. The publication of Chêne et al 2015 provided the best overview of the lifetime risk of dementia in the general population with data from the ages of 45 to 106 years [65]. It was used to create a line chart visualizing the change in risk of dementia over age (Fig 3). Second, a systematic search was conducted in four literature databases (PubMed, Web of Science, PsycInfo, Livivo) to identify a source for the incidence of dementia in biomarker-positive individuals. The search for full-text publications of the last ten years based on a wide search term (“incidence AND risk AND dementia AND amyloid AND positive”) revealed 182 search results. After screening, two publications were considered. Ossenkoppele et al 2022 published information on the different risks of future cognitive progression of cognitively healthy individuals with amyloid positive and amyloid negative positron emission tomography results [66]. To visualize the increased hypothetical risk, bar charts providing a general overview of all ages including information on a higher risk (biomarker positive) in comparison to a lower risk (biomarker negative) of progressing to dementia as well as pictograms on individual risks based on biomarker status and age were created (Fig 4). Previously published practice recommendations for communicating risk showed that the visualization of risk is a useful addition to numerical risks [30,67,68]. Single items of the questionnaire were developed in internal expert workshops.

### Summary

The PreTAD project systematically and comprehensively (1) learns about attitudes, needs, and perspectives on AD dementia risk estimation of the general population and cognitively unimpaired individuals with and without previous contact with memory clinics, (2) identifies anticipated impacts of AD dementia risk estimation, and (3) discusses the implications of the paradigm shift in medicine at individual and societal levels, from an ethical, linguistic, and legal perspective.

Therefore, different approaches are used: (1) an assessment in memory clinics, (2) an assessment of a general population without previous experience with dementia, and (3) an assessment of the general population via an online survey. The empirical findings are analyzed from a clinical, ethical, linguistic, and legal point of view. The trinational research design provides a basis for national comparability. The longitudinal approach helps to understand if attitudes, needs, and perspectives change over time and helps to understand the general challenges of a predictive turn in AD dementia on individual and societal levels. PreTAD contributes to creating a comprehensive framework for clinical practice for biomarker-based AD dementia risk estimation in preclinical stages.

## Discussion

Due to the development of disease-modifying therapies, advances in dementia risk estimation and prevention of AD dementia, personal attitudes, needs and perspectives of individuals and society are becoming increasingly important. Dementia risk estimation can form the basis for informed decision-making about preventive measures in case of an increased risk and for early action to potentially reduce the risk [67]. In addition to the further development of diagnostic methods, attention should be paid to the possible consequences of risk knowledge and psychological distress.

Health communication challenges in genetic testing for neurodegenerative diseases including specified physician training and patient education, have been highlighted before [69]. It is important to note the psychological impact of risk knowledge, e.g., the potential change in self-perception [29,70]. A systematic review showed a transient increase in levels of anxiety, depression, poorer perception of health, and psychological distress in those who tested positive for an increased risk of disease (for various diseases, e.g., cardiovascular risk, risk of AIDS, risk of cancer), without evidence for long-term effects [71]. If this turns out to be a typical risk of predictive testing, patients must be informed beforehand to meet legally binding requirements. From an ethical point of view, an encompassing and patient-centered counseling about the possible effects of risk knowledge on different life areas is required. Consideration of language and communication is currently limited to counseling and information materials, rarely considering the diversity of (communicative) possibilities [72,73].

The recommendations on counseling and disclosure for cognitively healthy individuals and those with SCD in the clinical setting are still underdeveloped. Previous publications highlight important research areas to be considered before the implementation of preclinical biomarker testing in routine clinical care [16]. Ethical aspects such as shared decision-making as well as the law, especially concerning the right to know and the right not to know, and the opportunity and time to reconsider one’s own decision should be given sufficient consideration [23]. Discussion of potential risks (including psychological distress) and potential benefits (e.g., implementation of preventive measures based on lifestyle, and future life planning) empowering patients to make an informed decision about AD dementia risk estimation is needed [74]. It can clarify how much information a doctor needs to provide before predictive testing, based on the patient’s wishes, which may go beyond the legal requirements, and protect physicians from liability risks due to a lack of provided information. Counseling should focus on comprehensive risk communication, enabling individuals to make informed decisions about their diagnostic pathway, to understand uncertainties, and to assert their right to self-determination [75,76]. It should contribute to a shared decision-making process that fosters good, trusting relationships between all parties involved [34]. Communication about dementia risks should be accurate and include a visual representation of absolute risks to support the shared decision-making process. Beyond this, a scoping review from Perry et al. highlighted the need for further research on the specific needs of individuals to determine the requirements for a multi-step counseling approach including pre-biomarker testing counseling, disclosure, and post-disclosure follow-up [77].

As predictive methods will likely become more accessible, new questions arise that touch clinically, ethically, and legally relevant aspects on both an individual and a societal level [28], including potential changes in self-perception [29], discrimination potentials, and (self-) stigmatization [70], the understanding of social contracts and principles in health care, including solidarity, financing, and regulatory instruments to ensure quality and freedom in medical treatment. Due to the complexity of biomarker-based dementia risk estimation, an ethical framework for the use of biomarker testing in routine clinical care, which raises awareness of conflicting ethical values, points out ways of resolving these conflicts in a conscientious manner, and contains specific recommendations for information and communication of results, is needed. To protect all parties involved, such a framework must be based on a legal analysis to ensure that the applicable law is respected at all times throughout the treatment process, even when new types of examinations such as AD dementia risk estimation are carried out, to avoid harm to the patient and liability for the medical profession.

Empirical research in this field can help to identify, illuminate, and understand individual and societal implications that go along with the predictive turn in AD dementia. Moreover, these insights can help to formulate recommendations for dealing with AD dementia risk estimation in clinical practice.

A main limitation of this project is its hypothetical nature, as the actual process of plasma biomarker testing and risk disclosure may affect the assessed topics differently. Another limitation is the exploratory character of this study with the use of newly developed questionnaires which are not yet validated in larger study samples.

The PreTAD project is currently in the recruitment phase. To our knowledge, it is the first study to investigate the impact of hypothetical biomarker-based AD dementia risk estimation on preclinical individuals, first-degree relatives of dementia patients, and the general population in a mixed-method approach.

## Supporting information

S1 File Checklist SPIRIT. (DOC)

S2 File Study protocol PreTAD German. (PDF)

S3 File Study protocol PreTAD English. (PDF)
