# Supplementary material for: Dementia risk estimation in persons at risk and the predictive turn in Alzheimer’s disease—The PreTAD project: Study protocol with an ethical, clinical, linguistic, and legal approach
Source: PLoS One. 2025 Jul 16;20(7):e0319868. doi: 10.1371/journal.pone.0319868 (PMC12266435; doi:10.1371/journal.pone.0319868)
Supplement: S2 File — (PDF) [file pone.0319868.s002.pdf]

Studienprotokoll Projekt: "Prädiktion der Alzheimer-Erkrankung: Ethische, klinische, linguistische und rechtliche Aspekte des Paradigmenwechsels einer prädiktiven Medizin“ (PreTAD)

Zusammenfassung des Projekts

Hintergrund: In der Medizin zeichnet sich heute ein Paradigmenwechsel ab, bei dem die auf Krankheitssymptomen basierende Diagnostik und Therapie durch Früherkennung, Risikovorhersage und Prävention ergänzt oder sogar ersetzt wird. Im Hinblick auf die Alzheimer-Krankheit (AD) werden die diagnostischen Methoden zur Früherkennung und Risikovorhersage ständig weiterentwickelt, wobei die diagnostische Genauigkeit zunimmt. Blutbasierte Biomarker könnten die klinisch angewandte, aber vergleichsweise invasive und teure Diagnostik (z. B. Liquor und PET) zum Nachweis der Alzheimer-Pathologie ersetzen. Die sich daraus ergebende höhere Verfügbarkeit kann dazu führen, dass die Risikovorhersage in diesem Zusammenhang verstärkt eingesetzt wird, z. B. unter Einbeziehung von Patienten mit subjektiver kognitiver Störung (SCD) oder symptomfreien Personen. Das PreTAD-Projekt zielt darauf ab, Daten zu den unterschiedlichen Sichtweisen der Menschen auf die Vorhersage der Alzheimer-Demenz sowie zu den Auswirkungen der prädiktiven Medizin auf den Einzelnen und die Gesellschaft aus klinischer, ethischer, rechtlicher und sprachlicher Sicht bereitzustellen. Das PreTAD-Projekt wird in Zusammenarbeit an drei Standorten (Köln/Bonn, Genf und Barcelona) durchgeführt.

Fragestellung: Die ethische Debatte über Risikovorhersagen auf individueller und gesellschaftlicher Ebene umfasst medizinische, kulturelle, rechtliche und philosophische Aspekte. Neu aufkommende blutbasierte Biomarker könnten den Zugang zur Alzheimer-Risikovorhersage erheblich erleichtern. Dies könnte den Wunsch und den Druck erhöhen, Alzheimer-Biomarker-Diagnostik nicht nur bei kognitiv beeinträchtigten, sondern auch bei präklinischen und asymptomatischen Personen durchzuführen. Als trinationales Gemeinschaftsprojekt soll die PreTAD-Studie dazu beitragen, einen Rahmen für eine gute klinische Praxis bei der Vorhersage von Alzheimer in präsymptomatischen und frühen (subjektiven) symptomatischen Stadien zu schaffen.

Methode: Wir beabsichtigen, die Rekrutierung auf insgesamt fünf verschiedene Gruppen von Proband:innen zu konzentrieren:

(a) 8-10 kognitiv gesunde Personen ohne familiäre Vorgeschichte oder persönliche Erfahrung mit Alzheimer-Demenz (nur von den deutschen Standorten durchgeführt), (b) 150 gesunde Teilnehmer:innen (50 Teilnehmer:innen pro Standort) mit einem potenziell höheren Risiko für die Entwicklung einer Alzheimer-Demenz aufgrund einer familiären Vorgeschichte von Alzheimer bei Verwandten ersten Grades oder aufgrund des Nachweises eines APOE4-Allels, (c) 150 Teilnehmer:innen (50 Teilnehmer:innen pro Standort) mit früherer SCD-Diagnose ≤ 2 Jahre und > 4 Wochen, (d) 90 Teilnehmer:innen (30 Teilnehmer:innen pro Standort) mit neuer SCD-Diagnose (≤ 4 Wochen) und (e) N≈1800 Personen aus der Allgemeinbevölkerung (≈1000 in Deutschland, ≈500 in Spanien, ≈300 in der Schweiz) (nur von den deutschen Standorten durchgeführt). Um einen umfassenden Ansatz zu erreichen, kombiniert die interdisziplinäre Zusammenarbeit eine quantitative Bewertung, qualitative (narrative) Interviews (Gruppen a-d, nur deutsche Standorte) und empirisch gestützte theoretische Ansätze zur ethischen, sozialen und rechtlichen Analyse.

Die Studienteilnehmer:innen werden mit einer quantitativen Testbatterie mit teilweise neu entwickelten Fragebögen, sowie hypothetischen Szenarien zur Offenlegung des Alzheimer-Biomarker-Befundes und Demenz-Risikos befragt. Die Gruppen a-d erhalten die gesamte Fragebogenbatterie, während die Gruppe e an einer Online-Querschnittserhebung teilnimmt und eine verkürzte Version der Fragebogenbatterie erhält, um die Benutzerfreundlichkeit zu erhöhen und eine repräsentative Stichprobengröße in jedem Land zu erreichen. Die Längsschnittuntersuchung (Gruppe b-d) wird von spezialisierten Zentren für Gedächtnisstörungen in Köln, Genf und Barcelona über einen Zeitraum von 12 Monaten durchgeführt.

Im Rahmen des qualitativen Ansatzes von PreTAD, der nur am deutschen Standort durchgeführt wird, werden insgesamt 40 narrative Interviews mit N=40 Teilnehmer:innen zu Beginn der Studie mit Personen aus den Gruppen a-d (n=8-10 pro Gruppe) durchgeführt. Den Studienteilnehmer:innen wird ein optionales Follow-up-Interview 1 Jahr nach dem Baseline-Interview angeboten. Wenn Proband:innen aus den Gruppen c + d von Angehörigen zum Interviewtermin begleitet werden, werden diese ebenfalls zur Teilnahme an den Interviews eingeladen.

In einer ersten Pilotstudie am deutschen Standort wurde die Durchführbarkeit der umfassenden Fragebogenbatterie für den quantitativen Ansatz mit N=20 Teilnehmer:innen (mit oder ohne diagnostizierter SCD) getestet. Für den qualitativen Ansatz wurden die neu entwickelten Interviewleitfäden mit n=8 Teilnehmer:innen der Gruppen a-d vorab getestet. Gekürzte Fragebögen für Gruppe e wurden mit N=24 Teilnehmer:innen getestet.

Ergebnisse und praktische Implikationen: Die von uns im Rahmen des klinisch-empirischen Ansatzes bereitgestellten Daten dienen der Klärung und Untersuchung der Perspektiven, Bedürfnisse und Präferenzen kognitiv gesunder Personen mit erhöhtem Risiko für die Entwicklung einer Alzheimer-Demenz und von Personen in präklinischen Stadien im Hinblick auf die Untersuchung auf frühe Alzheimer-Biomarker Diagnostik und die Vorhersage der Alzheimer-Demenz. Unsere Bewertung liefert Informationen über das allgemeine numerische Risikoverständnis und die Erwartungen an die Risikovorhersage. Sie konzentriert sich auch auf die psychische Belastung während des Prozesses der Risikovorhersage, das subjektive Wohlbefinden, die Lebensqualität (QoL), die Identität und die Selbstwahrnehmung, sowie die Bereitschaft zu Lebensstiländerung und Präventivmaßnahmen.

Verantwortlichkeiten

Studienleiterin dieses Projekts ist Prof. Dr. Christiane Woopen, Inhaberin der Heinrich-Hertz-Professur für Lebensethik, TRA4 an der Universität Bonn, Deutschland. An den deutschen Standorten wird die Studie am Zentrum für Lebensethik der Universität Bonn und am Zentrum für Gedächtnisstörungen der Klinik und Poliklinik für Psychiatrie und Psychotherapie des Universitätsklinikums Köln durchgeführt. In Köln ist Dr. Ayda Rostamzadeh für die Durchführung der quantitativen Befragung der Gruppen b-d verantwortlich. Die Verantwortlichkeit für die Rekrutierung der Proband:innengruppen b-d liegt ebenfalls am Zentrum für Gedächtnisstörungen der Uniklinik Köln. In Barcelona ist Prof. Dr. Boada, Gründer des Ace Alzheimer Center Barcelona, Universitat Internacional de Catalunya, für die Durchführung der quantitativen Erhebung der Gruppen b-d verantwortlich. In Genf ist Prof. Dr. Frisoni, Leiter der Abteilung für Rehabilitation und Geriatrie des Genfer Gedächtniszentrums am Universitätsspital Genf, für die Durchführung der Befragungen der Gruppen b-d an diesem Standort verantwortlich. Das Center for Life Ethics der Universität Bonn ist für die Rekrutierung der Teilnehmer:innen sowohl für die quantitative als auch für die qualitative Umfrage der Gruppe a (nur deutscher Standort) und der Gruppe e (Online-Umfrage, alle Standorte) verantwortlich. Das Projekt wird durch das ERA-NET NEURON (Network of European Funding of Neuroscience Research) und das Bundesministerium für Bildung und Forschung (BMBF) gefördert.

Die Eintragung dieses Studienprojekts in das deutsche Studienregister erfolgte nach Genehmigung durch die koordinierende Ethikkommission der Universität Bonn (DRKS-ID: DRKS00029035).

Wissenschaftlicher Hintergrund

Die gesellschaftlichen Auswirkungen eines Paradigmenwechsels hin zu einer prädiktiven Medizin sind bisher vergleichsweise selten Gegenstand der Forschung. Das Fehlen von Behandlungsmethoden der kognitiven Beeinträchtigung im Zuge einer Alzheimer-Krankheit (AD) führt zu einer erheblichen Ausweitung der prädiktiven Methoden mit dem Ziel einer immer früheren Vorhersage, weshalb dieser Bereich als ein sehr geeignetes Beispiel für die Untersuchung des „Predictive Turn" in der Medizin auf

verschiedenen Ebenen angesehen werden kann. Gleichzeitig sind neurodegenerative Erkrankungen, die Demenz verursachen, wie z. B. AD, zu einer großen Herausforderung für die öffentliche Gesundheit geworden. Dennoch sind Studien zu den Auswirkungen einer frühen Demenz-Vorhersage im Bereich der AD-Forschung noch selten und messen meist die Effekte auf individueller Ebene. Insgesamt wird jedoch die Dringlichkeit einer "Überprüfung der ethischen Rahmenbedingungen" (Angehrn et al., 2019) hervorgehoben.

Die AD als pathophysiologische Ursache der Alzheimer-Demenz, beginnt bereits Jahrzehnte bevor Symptome auftreten. Eine Früherkennung der AD in präklinischen und prodromalen Stadien ist bereits lange bevor die Funktionseinschränkungen einer Demenz einsetzen möglich (Jack et al., 2018), wobei die präklinische Phase der AD durch das Stadium der subjektiven kognitiven Störung beschrieben wird. Im Rahmen dieser wird eine anhaltende subjektive Beobachtung von kognitiven Einschränkungen im Vergleich zum individuellen Ausgangsniveau bemerkt. Während SCD zunächst ein unspezifisches und heterogenes Syndrom beschreibt, das bei älteren Personen nicht mit der AD assoziiert sein muss, zeigen jüngste Forschungsergebnisse, dass Personen mit SCD und einem Biomarker-Nachweis für eine AD ein erhöhtes Risiko für eine spätere kognitive Verschlechterung haben (Wolfsgruber et al., 2017; Tijms et al., 2017; Van Maurik et al., 2019; Ebenau et al., 2020.). Die vorhandenen klinischen Studiendaten deuten darauf hin, dass zahlreiche Hürden und Unsicherheiten in der Kommunikation von AD-Biomarker Ergebnissen bei kognitiv Gesunden bestehen. Diese resultieren aus dem möglicherweise unklaren konzeptuellen Verständnis für die prädiktive Diagnostik und der Einschätzung von Risikowahrscheinlichkeiten bei dieser Personengruppe, in Abgrenzung zu definitiven Diagnosen (Mozersky et al., 2018). Insbesondere bei der Kommunikation von einer Risikobestimmung besteht die Gefahr, dass ein Krankheitsrisiko mit einer Krankheitsdiagnose verwechselt wird (Milne et al., 2018). Weiterhin zeigten sich in Interviews mit kognitiv gesunden Proband:innen, die im Rahmen der Studie SOKRATES (Study of Knowledge and Reactions to Amyloid Testing) eine Amyloid-Positronenemissionstomographie-(PET)-Bildgebung erhielten, dass Sorgen hinsichtlich einer Stigmatisierung aufgrund pathologischer Befunde bestehen und die Testergebnisse bedeutsam für die Identität, Selbstbestimmung und sozialen Interaktionen der Befragten sind (Largent et al., 2020).

Während prädiktive Methoden immer sicherer, erschwinglicher und leichter zugänglich werden, ergeben sich daraus neue Fragen, die verschiedenste disziplinäre Domänen berühren. Ethisch und anthropologisch sind z. B. Veränderungen in der Selbstwahrnehmung des Menschen, Diskriminierungspotentiale und (Selbst-)Stigmatisierung zu berücksichtigen, sowie Einflüsse auf die Selbstbestimmung und die sozialen Beziehungen; soziologisch und ethisch geht es um das Verständnis von Sozialverträgen und Prinzipien in der Gesundheitsversorgung, einschließlich der Solidarität; sozioökonomisch und rechtlich sind die Veränderungen von Gesundheitssystemen und ihrer Finanzierung sowie ordnungspolitische Instrumente der Sicherung von Qualität und Freiheit in der medizinischen Behandlung neu zu bewerten. Dazu zählen ebenfalls medizinische Indikationen, Information und Einwilligung sowie Versorgungsstandards und Haftung.

Insbesondere bei der Kommunikation von einer Risikobestimmung im Rahmen einer Früherkennungsuntersuchungen besteht die Gefahr, dass ein Krankheitsrisiko mit einer Krankheitsdiagnose verwechselt wird. Hier konnte gezeigt werden, dass bei kognitiv gesunden Personen das konzeptuelle Verständnis für die prädiktive Diagnostik und von Risikowahrscheinlichkeiten, in Abgrenzung zu definitiven Diagnosen, eine Herausforderung darstellt (Mozersky et al., 2018; Milne et al. 2018). Die Betrachtung von Sprache und Kommunikation im Rahmen der klinischen AD-Forschung beschränkt sich zurzeit auf die Erstellung von Beratungs- und Informationsmaterialien (Samerski/ Henkel (2015); Samerski (2015)). Nur selten wird die Vielfalt der (kommunikativen) Möglichkeiten der individuellen Risikowahrnehmung und -verarbeitung oder auch

die Reflexion von habitualisierten kommunikativen Praktiken und sprachlichen Mustern im Bereich der prädiktiven Medizin und der Risikokommunikation berücksichtigt (Lorke et al. 2021).

Blickt man auf die Bedarfe in der klinischen Praxis, gibt es erste Publikationen zu Handreichungen für kognitiv gesunde Studienproband:innen, die an AD-Früherkennungsuntersuchungen teilnehmen (Mozersky et al., 2021). Obwohl kürzlich veröffentlichte Empfehlungen zu Liquor- und PET-basierten AD-Biomarker Untersuchungen einen Rahmen für die AD-Diagnostik ab dem symptomatischen Stadium der AD bieten, fehlen Empfehlungen zur Beratung und Befundübermittlung für kognitiv Gesunde sowie Personen mit SCD (Harkins et al., 2015; Simonsen et al., 2017; Johnson et al., 2013; Shaw et al., 2018).

Besonders aufgrund der schweren Belastung, die mit der AD verbunden ist, und der starken Präsenz der Krankheit im medialen Diskurs steigt die Hoffnung auf eine wirksame Behandlung in der Zukunft. Durch die aufkommenden Möglichkeiten der Bestimmung von Biomarkern aus dem Blut könnten der Wunsch und die Nachfrage, AD-Biomarkeruntersuchungen bei asymptomatischen Personen und Personen mit SCD durchzuführen, steigen. Dies erhöht die Dringlichkeit der o.g. Forschungsfragen und des interdisziplinären und multiperspektivischen Ansatzes dieses Projekts.

Projektziele

Das übergreifende Ziel von PreTAD ist es, individuelle Bedürfnisse und unterschiedlichen Perspektiven der Menschen in Bezug auf die Vorhersage von AD herauszuarbeiten sowie die Auswirkungen des Paradigmenwechsels in der Medizin auf individueller, sprachlicher, rechtlicher und gesellschaftlicher Ebene zu erörtern.

Um zu klären, welche Bedürfnisse in Bezug auf Gesundheitssysteme und ihre Angebote individuell und gesellschaftlich vorliegen, welche unterschiedlichen Perspektiven auf die Prädiktion eingenommen werden und welche spezifischen Entscheidungsparameter (für oder gegen die Prädiktion) dabei eine Rolle spielen, sollen im Rahmen der individuellen Befragungen sowohl Personen mit SCD als auch Personen mit Familienanamnese von AD oder APOE4-Allel-Träger:innen sowie gesunde Personen (Allgemeinbevölkerung) in die Studie inkludiert werden. Zusätzlich wird die diskursive, öffentlich-mediale Perspektive untersucht, um das gesellschaftliche Meinungsbild in die Analyse zu integrieren.

Durch die Untersuchung der Auswirkungen der prädiktiven Medizin auf das Individuum, die jeweilige Gesellschaft und das Gesundheitssystem am Beispiel von AD, sollen schlussendlich ein Rahmenwerk und Richtlinien für die präsymptomatische Vorhersage der Alzheimer-Demenz entwickelt werden, welche für die klinische Praxis hilfreich sein kann. Zusätzlich werden die Erkenntnisse aus PreTAD im Projektverlauf in wissenschaftlichen Fachpublikationen (open access) reflektiert und öffentlich zugänglich gemacht, sodass diese einen Beitrag zur wissenschaftlichen Diskussion in den entsprechenden Fachgebieten leisten.

Zielgrößen

In einem Mixed-Methods-Forschungsdesign werden in PreTAD qualitative und quantitative Daten zur Auswertung der erhobenen Daten kombiniert.

Im ethischen Teilprojekt von PreTAD wird ein Mixed-Methods-Ansatz angewendet, der aus der Kombination der Ergebnisse der statistischen Erhebungsanalyse (quantitative Daten) und einer strukturierten thematischen Analyse der qualitativen Daten beruht, die zu ethischen Bewertungen und typischen Differenzierungen führt. Die Auswertung der Interviewdaten erfolgt über das Analysetool 'MAXQDA'.

Die im Rahmen des klinischen Teilprojekts von PreTAD erhobenen empirischen Daten ermöglichen eine länderübergreifende Vergleichbarkeit. Die in diesem Teilprojekt eingesetzten statistischen Methoden umfassen parametrische Ansätze des Gruppenvergleichs (z.B. T-Statistik, Varianzanalyse) sowie Regressionsanalysen zur Assoziationstestung.

Für das linguistische Teilprojekt von PreTAD werden die transkribierten Interviews aufgrund der engen Verzahnung mit dem ethischen Teilprojekt ebenfalls mit dem Analysetool ‚MAXQDA‘ analysiert. In diesem Teilprojekt wird als Analyseverfahren der Interviews die linguistische Konversationsanalyse durchgeführt. Diese ermöglicht eine Fokussierung auf das Handeln mit Sprache und angewendete kommunikative Praktiken.

In der Analyse des rechtlichen Teilprojektes von PreTAD werden klassische Methoden der Systemanalyse, der Rechtsauslegung, der Interpretation von Regelungen, der Schaffung von Regelungslenkungen und -vorgaben, des Rechtsvergleichs, insbesondere unter Berücksichtigung der Systeme der Schweiz, Spaniens und Deutschlands, und dem Aufzeigen von Knackpunkten mit Regelungs- oder politischem Handlungsbedarf angewendet.

Studienpopulation

Übersicht:

Teilnehmer:innen	• Gruppe a: 8-10 kognitiv gesunde Personen ohne familiäre Vorgeschichte oder persönliche Erfahrung mit Alzheimer-Demenz (nur Deutschland)<br>• Gruppe b: 150 kognitiv gesunde Personen mit positiver Familienanamnese oder APOE4-Status (n=50 pro Standort)<br>• Gruppe c: 150 Personen mit subjektiver kognitiver Beeinträchtigung (SCD) (vorherige SCD-Diagnose > 4 Wochen und ≤ 2 Jahre) (n=50 pro Standort)<br>• Gruppe d: 90 Personen mit SCD (neue SCD-Diagnose ≤ 4 Wochen) (n= 30 pro Standort)<br>• Gruppe e: 1.800 Personen aus der Allgemeinbevölkerung (in Deutschland ≈ 1.000, Spanien ≈ 500, Schweiz ≈ 300)

Einschluss- und Ausschlusskriterien:

Allgemeine Einschlusskriterien (Umfrage und Interviews)	• Mindestens 18 Jahre alt<br>• Ausreichende Beherrschung der Landessprache des jeweiligen Studienortes, so dass eine informierte Zustimmung gegeben und das Ausfüllen der Fragebögen gewährleistet werden kann<br>• Nur Interviews: ausreichende Beherrschung der deutschen Sprache, so dass die qualitativen Interviews in deutscher Sprache durchgeführt werden können
Allgemeine Ausschlusskriterien (Umfrage und Interviews)	• Unzureichende Kenntnisse der Landessprache<br>• Analphabetismus<br>• Personen, die nicht einwilligungsfähig sind oder keine schriftliche Einwilligung gegeben haben

Zusätzlich zu den allgemeinen Ein- und Ausschlusskriterien gelten folgende gruppenspezifische Kriterien:

Gruppe a (kognitiv gesunde Personen ohne familiäre Vorgeschichte oder persönliche Erfahrung mit Alzheimer-Demenz (nur Deutschland)):

Zusätzliche Einschlusskriterien	Keine
Zusätzliche Ausschlusskriterien	• Subjektive oder objektive kognitive Beeinträchtigung<br>• Mittelschwere bis schwere depressive Störung; Hospital Anxiety and Depression Scale (HADS) Score > 10 Punkte<br>• Enger Kontakt zu einer Person mit Alzheimer-Krankheit<br>• (Vorheriger) Besuch eines Alzheimer-Präventionszentrums oder einer vergleichbaren Einrichtung<br>• Personen mit bekanntem ApoE-e3/e4- oder ApoE-e4/e4-Genträgerstatus

Gruppe b (Teilnehmer:innen mit Verwandten ersten Grades, bei denen Alzheimer diagnostiziert wurde oder die Träger des ApoE4-Gens sind):

Zusätzliche Einschlusskriterien	• Kenntnis einer medizinischen Diagnose der Alzheimer-Krankheit bei mindestens einem Verwandten ersten Grades (Mutter, Vater, Geschwister) oder<br>• Teilnehmer:innen mit einem ApoE-e3/e4- oder ApoE-e4/e4-Genträgerstatus
Zusätzliche Ausschlusskriterien	• Subjektive oder objektive kognitive Beeinträchtigung<br>• Mäßige bis schwere depressive Störung; Hospital Anxiety and Depression Scale (HADS) Score > 10 Punkte

Gruppe c-d (Teilnehmer:innen mit SCD-Diagnose > 4 Wochen und ≤ 2 Jahre (Gruppe c) und ≤ 4 Wochen (Gruppe d)):

Zusätzliche Einschlusskriterien	• Klinische Kriterien für die Diagnose von SCD (gemäß den Kriterien von Jessen et al. 2014) > 4 Wochen und ≤ 2 Jahre (Gruppe c) oder ≤ 4 Wochen (Gruppe d)<br>• Eine subjektive und anhaltende (nicht akute) Verschlechterung der kognitiven Leistung im Vergleich zum ursprünglichen Ausgangsniveau, die nicht auf ein akutes Ereignis zurückzuführen ist<br>• Neuropsychologische Testbatterie, die für leichte kognitive Beeinträchtigung (MCI) oder prodromale Alzheimer-Erkrankung verwendet wird, zeigt ein Ergebnis innerhalb der alters-, geschlechts- und bildungsbereinigten Normgruppe
Zusätzliche Ausschlusskriterien	• MCI, Prodromal-AD oder Demenz<br>• Beeinträchtigungen können durch eine psychiatrische* oder neurologische Erkrankung (außer Alzheimer), somatische Erkrankung, Medikamente oder Substanzmissbrauch erklärt werden<br>• Mäßige bis schwere depressive Störung; HADS-Score > 10 Punkte

	*Milde subsyndromale depressive Symptome oder Angstsymptome gelten nicht als Ausschlusskriterien.

Gruppe e (Personen aus der Allgemeinbevölkerung):

Zusätzliche Einschluss-/Ausschlusskriterien	Keine

Für die Teilnehmer:innen an den Pilotstudien gelten die gleichen Ein- und Ausschlusskriterien.

Rekrutierung

Die Rekrutierung für die Fragebogenstudie ist wie folgt geplant:

Gruppe a (kognitiv gesunde Allgemeinbevölkerung ohne familiäre Vorbelastung oder persönliche Erfahrung mit Alzheimer-Demenz, nur deutscher Standort)

Die Teilnehmer:innen der Gruppe a werden über (soziale) Medien rekrutiert. Darüber hinaus kann auch die Schneeballsystem-Strategie (Teilnehmer:innen empfehlen interessierte Bekannte oder Freunde) zur Rekrutierung genutzt werden. Die Befragung und das Interview werden persönlich an einem der deutschen Studienstandorte oder per Zoom durchgeführt. Vor der Befragung und dem Interview werden die Teilnehmer:innen über den Zweck der Studie sowie die Verwendung und Speicherung der Daten informiert. Anschließend müssen die Teilnehmer:innen ihre schriftliche Zustimmung zur Teilnahme an der Studie geben.

Gruppe b (Teilnehmer:innen mit Verwandten ersten Grades, bei denen Alzheimer diagnostiziert wurde oder die ApoE4-Genträger sind)

Die Rekrutierung der Proband:innen aus Gruppe b erfolgt über das Kölner Alzheimer Präventionsregister. Die Kontaktaufnahme erfolgt über eine Rundmail mit einem Hinweis auf die PreTAD-Studie. Dieser Mail werden Kontaktinformationen zur Studienteilnahme beigefügt.

Gruppe c und d (Teilnehmer:innen mit SCD-Diagnose > 4 Wochen und ≤ 2 Jahre (Gruppe c) und ≤ 4 Wochen (Gruppe d))

Die Teilnehmer:innen der Gruppen c und d in Deutschland werden über das Zentrum für Gedächtnisstörungen an der Uniklinik Köln durch persönliche Ansprache rekrutiert.

Gruppe e (Personen aus der Allgemeinbevölkerung):

Die Teilnehmer:innen der Gruppe e werden über einen externen Dienstleister rekrutiert, der auf die Durchführung von groß angelegten Umfragen für den akademischen Bereich spezialisiert ist.

Der Studienplan für alle Studienarme ist unten dargestellt:

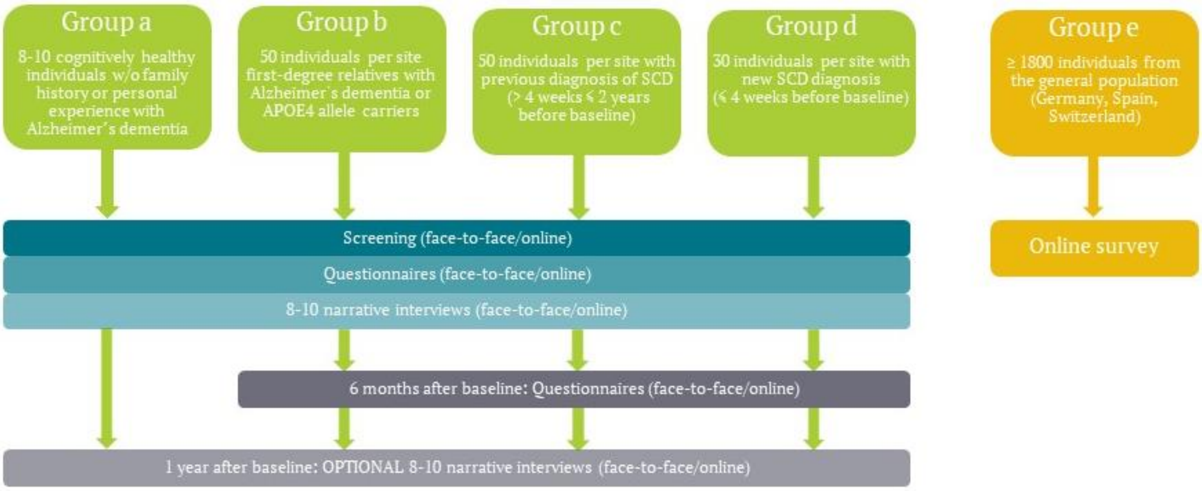

Abbildung 1 Studien- und Rekrutierungsplan PreTAD

Methodik und Durchführung

Bei diesem Projekt handelt es sich um ein trizentrisches Studienprojekt. Das Projekt wird in Deutschland (Bonn, Köln), der Schweiz (Genf) und Spanien (Barcelona) durchgeführt. Die Interviewstudie wird nur in Deutschland durchgeführt.

PreTAD wendet sich an fünf Teilnehmer:innengruppen, um die differenzierte Sicht von gesunder Bevölkerung mit verschiedenen Arten von Kontakt zu Gedächtnisambulanzen und Alzheimer-Demenz (Gruppen a-d) und der Allgemeinbevölkerung (Gruppe e) zu berücksichtigen. Alle potenziellen Teilnehmer:innen erhalten Informationsblätter zur Studie, in denen die Ziele der Studie, der genaue Studienablauf, Informationen zur Freiwilligkeit und Pseudonymisierung der Daten, Informationen zu neuen Erkenntnissen, Informationen zur Teilnehmer:innenversicherung (Gruppe a-d) und die Kontaktdaten des Studienzentrums vermerkt sind. Wenn sie sich dann für die Teilnahme an der Studie entscheiden, erhalten die Teilnehmer:innen eine Einverständniserklärung, in der sie sich mit der Studie und den Datenschutzbestimmungen einverstanden erklären. Die Einverständniserklärung muss zu Beginn der jeweiligen Studie vorliegen.

Die Informationen und Einverständniserklärungen für die Online-Befragung der Allgemeinbevölkerung (Gruppe e) werden auf der ersten Seite des Befragungstools angezeigt und können durch Anklicken bestätigt werden.

Das Projekt gliedert sich in zwei Studienteile: In der Fragebogenerhebung werden Themen wie Lebensstil, Wissen über Vorhersage und Risiko sowie Fragen zu den Bedürfnissen und Perspektiven gegenüber der Alzheimer-Demenz-Vorhersage/neuen Testverfahren (Überlegungen/Parameter) abgefragt. Zusätzlich werden Fragen zur Einschätzung hypothetischer Szenarien der Risikokommunikation zur Alzheimer-Demenz einbezogen. In der Interviewstudie werden narrative Interviews durchgeführt, um zusätzliche Aspekte und Facetten der individuellen Perspektiven auf die Krankheitsprädiktion bei kognitiv gesunden Personen mit unterschiedlichen Vorerfahrungen mit dem Thema zu erheben (Gruppe a-d). Zehn Teilnehmer:innen pro Gruppe (a-d) werden in die Interviewstudie einbezogen.

Nur deutsche Standorte: Teilnehmer:innen, die an der Interviewstudie teilnehmen (Gruppen b-d), erhalten die Informationen und Einverständniserklärungen vom Studienarzt, wenn sie oder die

jeweiligen Familienmitglieder ihren regulären Termin im Kölner Alzheimer-Präventionszentrum wahrnehmen. Sollten potenzielle Studienteilnehmer:innen aus Gruppe b angeben, dass ein persönlicher Termin nicht in Frage kommt, bietet das Forschungsteam die Möglichkeit, das Interview per Zoom durchzuführen, wie bei Gruppe a, die alle notwendigen Unterlagen per E-Mail erhält.

Fragebogenstudie / Online-Umfrage:

Bei der quantitativen Erhebung (Fragebogenstudie, Gruppen a-d) werden sowohl Einstellungen und Bedürfnisse als auch Vorbehalte der Proband:innen gegenüber neuen prädiktiven Möglichkeiten am Beispiel der Alzheimer-Demenz mit Hilfe von Fragebögen untersucht. Wir werden validierte Fragebögen verwenden, die in der klinischen Psychologie weit verbreitet sind und Teil der regelmäßigen Beurteilung von Patienten in Gedächtniskliniken sind. Darüber hinaus werden wir neu entwickelte Fragebögen verwenden, die die Ziele der Studie im Hinblick auf die individuellen Einstellungen und Perspektiven zur Vorhersage der Alzheimer-Demenz widerspiegeln.

Die Teilnehmer:innen der Gruppen b-d werden auf die Möglichkeit der Studienteilnahme vor Ort im Zentrum für Gedächtnisstörungen (Klinik und Poliklinik für Psychiatrie und Psychotherapie der Uniklinik Köln) oder über das Alzheimer-Präventionsregister aufmerksam gemacht. Der Zeitaufwand für die Teilnehmer:innen beträgt insgesamt 45 Minuten für die Screening-Untersuchung und ca. 90 Minuten für die Baseline- und Abschlussuntersuchung (6-Monats-Follow-up).

Die verkürzte Version der Fragebogenbatterie für die Allgemeinbevölkerung (Gruppe e) hat eine Dauer von ca. 15 Minuten. Die folgenden Instrumente, von denen einige validiert, aber auch neu entwickelt wurden, sollen in PreTAD eingesetzt werden:

Fragebogen	***Gruppen a-d***	***Gruppe e***
Vorbestehend		
Mini Mental State Examination (MMSE) (Screening-Instrument)	X	-
Hospital Anxiety and Depression Scale (HADS) (Screening-Instrument)	X	-
SCD-Fragebogen (Screening-Instrument)	X	-
Fragebogen zum Lebensstil	X	Nur ein Item
Fragebogen zur Lebenszufriedenheit (FLZM)	X	-
Subjektives Zahlenverständnis	X	-
Objektives Zahlenverständnis	X	-
Einsamkeit (UCLA)	X	-
Brief Resilience Scale (BRS)	X	X
Big-Five-Inventar	X	X
Für PreTAD neu entwickelt		
Demografische Daten	X	Gekürzte Version
Frühere Erfahrungen mit Demenz	X	Gekürzte Version
Bedürfnisse und Entscheidungsparameter in Bezug auf Alzheimer-Screening und neue Testmethoden	X	Gekürzte Version
Subjektive Risikowahrnehmung	X	Gekürzte Version
Bewertung hypothetischer Szenarien von AD-Biomarkern: Befundberichterstattung und Risikovorhersage	X	Gekürzte Version (ohne Graphiken)

Tabelle 1: Übersicht über die Testbatterie für alle Gruppen.

Die quantitativen Daten der Fragebogenstudie werden in REDCap erfasst und gespeichert (Gruppen a-d). REDCap ist eine browserbasierte, metadatengesteuerte EDC-Software. Die Daten werden nicht auf

einem zentralen Server gespeichert, sondern auf dem Server der Institution, die die Software einsetzt (hier: Universität zu Köln). Alle Projektdaten werden von der lokalen Einrichtung gespeichert und gehostet und nicht an andere Einrichtungen oder Organisationen weitergeleitet. Da keine internationalen Server außerhalb der EU involviert sind, können mit REDCap die Anforderungen der EU-Datenschutzgrundverordnung (GDPR) erfüllt werden. Die Rechte zum Hinzufügen, Ändern oder Löschen von Daten können vom Administrator individuell vergeben werden.

Die quantitativen Daten der Gruppe e werden mit den GDPR-konformen Richtlinien des entsprechenden externen Dienstleisters verarbeitet.

Interview-Studie:

Zur Auswertung der Interviews, die am deutschen Studienstandort geführt werden, werden diese mit Zustimmung der Proband:innen mit einem Audiogerät aufgezeichnet und für die weitere Datenanalyse durch einen Dienstleister transkribiert. Die Interviews werden mittels einer qualitativen Inhaltsanalyse nach Kuckartz (2016) ausgewertet.

Das voraussichtliche Ende der Datenerhebung ist November 2023. Im Zeitraum der Interviewstudie werden die ersten Interviews transkribiert und sukzessive ausgewertet.

Vorstudie:

Am Standort Köln wurde dem quantitativen und qualitativen Studienarm zwischen Januar und Februar 2023 eine Vorstudie vorgeschaltet, um die neu entwickelten Fragebögen mit n=10 gesunden Personen und n=10 Proband:innen mit SCD zu testen. Dabei wurden die Fragebögen auf ihre Durchführbarkeit und Verständlichkeit überprüft. Die quantitativen Piloterhebungen wurden in Papierform durchgeführt und dauerten etwa 60 Minuten. Der Interviewleitfaden für die qualitative Studie wurde ebenfalls im Rahmen der Vorstudie mit n=2 Gesunden und n=6 Proband:innen mit SCD auf Machbarkeit getestet und dauerte ca. 60 Minuten.

Zwischenauswertung und Abbruchkriterien

Den Proband:innen wird jederzeit die Möglichkeit eingeräumt, die Teilnahme an der Studie ohne Angabe von Gründen abzubrechen oder die Beantwortung einzelner Fragen zu verweigern. Die Proband:innen werden darüber aufgeklärt, dass ihnen dadurch keinerlei Nachteil entsteht. Zeigen sich im Studienverlauf bei einem/r Proband:in die Einschlusskriterien nicht mehr erfüllt, wird die Studienteilnahme der/des Proband:in und der/des Angehörigen abgebrochen.

Ein vorzeitiges Ende der Studie tritt nur dann in Kraft, wenn die grundsätzlichen Gegebenheiten, die zur Bewilligung der Studiendurchführung, seitens der Ethikkommission oder auch des BMBF, widerrufen werden

Nutzen-Risiko-Abwägung

Da es sich um eine nicht-interventionelle Studie handelt, sind keine Komplikationen oder Risiken zu erwarten.

Es sind grundsätzlich die Regeln für die Befragung Erwachsener zu beachten (ADM 2021). Das Design dieser Studie lässt aus forschungsethischer Sicht auf keine Bedenken hinsichtlich einer durch die Teilnahme an dieser Studie herbeigeführten emotionalen Belastung schließen.

Es kommt zu keinem unmittelbaren Nutzen für die Teilnehmer:innen dieser Studie, jedoch kann die Reflexion über die Prädiktion von Krankheiten im Allgemeinen und der Prädiktion einer Alzheimer-

Demenz im Speziellen durch die Teilnahme evoziert werden, und eine Sensibilisierung für dieses Thema könnte eine positive Konsequenz der Teilnahme sein. Eine Teilnahme könnte hingegen auch negative Konsequenzen wie z.B. Ängste oder Unsicherheiten in der Auseinandersetzung mit dieser Thematik im Hinblick auf die eigene Gesundheit bei den Proband:innen auslösen. Grundsätzlich erfordert die Durchführung quantitativer und qualitativer Studiendesigns sorgsame und kontinuierliche Reflexionsleistungen der Forscher:innen, um dem Prinzip der Nicht-Schädigung von Teilnehmer:innen gerecht zu werden. Dies betrifft die Aspekte der Integrität der Forschenden, der Nicht-Täuschung der Studienteilnehmer:innen, des informierten Einverständnisses und der Freiwilligkeit der Teilnahme, der Schadensvermeidung vor, während und nach der Durchführung der Studie sowie der Vertraulichkeit und der Pseudonymisierung der Ergebnisse (von Unger 2014; Hopf 2016).

Es wird sichergestellt, dass die Proband:innen dieser Studie im Vorweg umfassend aufgeklärt und damit zu einem informierten Einverständnis befähigt wurden. Zudem ist die Freiwilligkeit der Teilnahme zu jeder Zeit gegeben und Proband:innen werden darüber informiert, dass sie jederzeit ein Anrecht auf das Zurückziehen der Einwilligung haben, ohne, dass dies nachteilige Konsequenzen für sie haben würde. Ebenso ist die Vertraulichkeit der Ergebnisse gewährleistet, im Falle einer Veröffentlichung sind diese in anonymisierter Form gemäß den Datenschutzrichtlinien zu publizieren.

Weder bei der quantitativen Erhebung noch bei der qualitativen Erhebung kann ausgeschlossen werden, dass sensible oder belastende Themen im Zusammenhang mit medizinischen prädiktiven Maßnahmen oder Themen der Alzheimer Demenz zur Sprache kommen. Konkrete Maßnahmen, um dieses Risiko zu minimieren, sind zu wiederholende Hinweise, dass die Proband:innen vollkommen selbstbestimmt entscheiden können, was, wie viel und worüber sie sich äußern. Belastungen für die Proband:innen oder mögliche Risiken vor, während und nach der Datenerhebung können in dieser Studie als insgesamt gering betrachtet werden.

Online-Umfrage/Fragebogenstudie:

Da die Fragen abwechslungsreich gestaltet sind und die Fragebögen von den Befragten auch zu einem späteren Zeitpunkt in Gruppen e und a (Online-Befragung), d.h. zu Hause oder an einem Ort ihrer Wahl, ausgefüllt werden können, ist die Dauer der Studie als nicht belastend zu erwarten. Das Ausfüllen der Fragebögen für die Gruppen b-d wird von geschultem Personal begleitet. Eine emotionale Belastung ist unwahrscheinlich, aber einige Fragen können für die Befragten kognitiv anspruchsvoll sein.

Interviewstudie:

Eine emotionale Belastung wird als unwahrscheinlich angesehen. Dennoch könnten einzelne Alltagssituationen im Zusammenhang mit der Vorhersage der Alzheimer-Demenz, die durch die Teilnahme an der Studie sensibler wahrgenommen werden, eine psychische Belastung darstellen. Ebenso könnte die geplante Dauer der Interviewstudie von 90 Minuten von einzelnen Proband:innen als zeitlich belastend empfunden werden. Wenn das Einverständnis gegeben ist, nehmen die Proband:innen an dieser Interviewstudie teil.

Biometrie

Da es sich bei PreTAD in erster Linie um eine explorative Studie handelt, wird für die Endpunkte mit erwarteten Effektgrößen in den Gruppen a-d keine Stichprobenberechnung durchgeführt. Die geplanten Analysen konzentrieren sich insbesondere auf die Unterschiede zwischen den Geschlechtern der Proband:innen und den verschiedenen Studienpopulationen sowie auf die transnationale Vergleichbarkeit der Studienländer. Es werden parametrische Ansätze des Gruppenvergleichs (z.B. T-Statistiken, Varianzanalysen) und Regressionsanalysen zur Assoziationsprüfung angewendet. Für die Gruppe e wird Repräsentativität angestrebt und eine Powerberechnung durchgeführt.

Die an deutschen Standorten erhobenen qualitativen Interviewdaten werden mit dem Analysetool MAXQDA ausgewertet und einer strukturierten thematischen Analyse nach Kuckartz (2017) unterzogen.

Datenmanagement und Datenschutz

Interviewstudie:

Sollten aus Sicherheitsgründen (z.B. Covid-Pandemie) zu einem späteren Zeitpunkt Kontaktbeschränkungen bestehen, wird die Interviewstudie mit allen Gruppen (a-d) online durchgeführt. Die Online-Durchführung der Interviewstudie erfolgt über das Videokonferenztool "Zoom" (https://rrzk.uni- koeln.de/support-information/informationen-zu-tools-fuer-kollaboratives-arbeiten/zoom-datenschutz-und-nutzungsvorgaben-fuer-hosts-moderatorinnen), das von der Universität zu Köln genutzt wird.

Der Grund dafür ist, dass der Dienstanbieter in der deutschen Bevölkerung bekannt, einfach zu bedienen und sehr effizient ist. Alle Teilnehmerinnen und Teilnehmer erhalten einen Link zur Teilnahme an der Interviewstudie mit entsprechenden Passwörtern. Zur Dokumentation und Auswertung der Interviews werden die Audios mit einem Aufnahmegerät des Olympus Audiosystems aufgezeichnet, so dass die Daten ausschließlich unabhängig vom Dienstleister "Zoom" lokal auf den Servern der Universität zu Köln zur Auswertung gespeichert werden. Zu Beginn der Studie füllen alle Befragten einen Vorab-Fragebogen zu ihren soziodemographischen Daten aus und senden diesen zusammen mit der Einverständniserklärung per Post an das Forschungsteam zurück

Interviewstudie und Online-Umfrage:

Alle persönlichen Daten werden in tabellarischer Form ausschließlich zum Zweck der Kontaktaufnahme und Bearbeitung der Studie gespeichert. Den Teilnehmer:innen wird eine Studien-ID zugewiesen. Diese Pseudonymisierungsliste wird getrennt von den erhobenen Daten auf dem lokalen Server der Universität zu Köln für die Dauer der Datenauswertung gespeichert. Nur der Studienleiter, der Studienkoordinator und die wissenschaftlichen Mitarbeiter des PreTAD-Forschungsteams haben Zugriff auf die Pseudonymisierungsliste. Im Datenmaterial selbst werden nur die Studien-IDs verwendet. Die Befragten werden darüber informiert, dass ihre Daten nur in pseudonymisierter Form verarbeitet werden und, dass die beteiligten Forscher zur Verschwiegenheit über persönliche Informationen verpflichtet sind. Die erhobenen Daten werden ausschließlich in pseudonymisierter Form für wissenschaftliche Forschungszwecke verwendet und nur in anonymisierter Form an die am Projekt beteiligten Länder (Schweiz und Spanien) weitergegeben. Die reinen Tonspuren der Interviewstudie werden zur Transkription an den Dienstleister weitergegeben. Die Befragten werden über diese Weitergabe informiert. Die Daten werden nicht an andere Dritte weitergegeben. Nur anonymisierte Daten werden für wissenschaftliche Publikationen verwendet. Die Proband:innen können ihre Teilnahme an der Studie jederzeit ohne Konsequenzen beenden und ihre Zustimmung zur Datennutzung widerrufen. In diesem Fall werden alle von dieser Person erhobenen Daten gelöscht. Die geltenden Datenschutzbestimmungen gemäß GDPR werden eingehalten. Das darin enthaltene Recht auf "Vergessen" durch Löschung der Daten ist gewährleistet.

Literaturverzeichnis

Angehrn, Z., Nordon, C. und Turner, A. (2019): Ethical and social implications of using predictive modeling for Alzheimer's disease prevention: a systematic literature review protocol. BMJ Open. 9, e026468.

Corbin, J. M. und Strauss, A. (1990). Grounded theory research: Procedures, canons, and evaluative criteria. Qualitative sociology, 13(1), 3-21.

Ebenau, J. L., Timmers, T., Wesselman, L., Verberk, I., Verfaillie, S., Slot, R., van Harten, A. C., Teunissen, C. E., Barkhof, F., van den Bosch, K. A., van Leeuwenstijn, M., Tomassen, J., Braber, A. D., Visser, P. J., Prins, N. D., Sikkes, S., Scheltens, P., van Berckel, B. und van der Flier, W. M. (2020): ATN classification and clinical progression in subjective cognitive decline: The SCIENCe project. Neurology, 95(1), e46–e58.

Flick, U. (2019): Qualitative Sozialforschung. Eine Einführung. Originalausgabe, 9. Auflage. Reinbek bei Hamburg: rowohlts Enzyklopädie im Rowohlt Taschenbuch Verlag (Rororo Rowohlts Enzyklopädie, 55694).

Harkins, K., Sankar, P., Sperling, R., Grill, J. D., Green, R. C., Johnson, K. A. und Karlawish, J. (2015): Development of a process to disclose amyloid imaging results to cognitively normal older adult research participants. Alzheimer's research & therapy, 7(1), 1-9.

Hopf, C. (2016): Forschungsethik und qualitative Forschung. In: Schriften zu Methodologie und Methoden qualitativer Sozialforschung. Springer VS, Wiesbaden. S. 195-205.

Jack Jr, C. R., Bennett, D. A., Blennow, K., Carrillo, M. C., Dunn, B., Haeberlein, S. B. und Silverberg, N. (2018): NIA-AA research framework: toward a biological definition of Alzheimer's disease. Alzheimer's & Dementia, 14(4), 535-562.

Johnson, K. A., Sperling, R. A., Gidicsin, C. M., Carmasin, J. S., Maye, J. E., Coleman, R. E. und AV45-A11 study group. (2013): Florbetapir (F18-AV-45) PET to assess amyloid burden in Alzheimer's disease dementia, mild cognitive impairment, and normal aging. Alzheimer's & Dementia, 9(5), 72-83.

Kuckartz, U. (2017): Computergestützte Inhaltsanalyse. In: Lothar Mikos und Claudia Wegener (Hg.): Qualitative Medienforschung. Ein Handbuch. 2., völlig überarbeitete und erweiterte Auflage. Konstanz, München: UVK Verlagsgesellschaft mbH; UVK Lucius (utb Medien- und Kommunikationswissenschaft, Pädagogik, Psychologie, Soziologie, 8314), S. 503–515.

Largent, E. A., Harkins, K., van Dyck, C. H., Hachey, S., Sankar, P., und Karlawish, J. (2020): Cognitively unimpaired adults' reactions to disclosure of amyloid PET scan results. PLoS One, 15(2), e0229137.

Lorke, M., Schwegler, C., & Jünger, S. (2021). Re-claiming the power of definition—The value of reflexivity in research on mental health at risk. In Qualitative Research Methods in Mental Health (pp. 135-165). Springer, Cham.

Milne, R., Bunnik, E., Diaz, A., Richard, E., Badger, S., Gove, D. und Brayne, C. (2018): Perspectives on communicating biomarker-based assessments of Alzheimer's disease to cognitively healthy individuals. Journal of Alzheimer's Disease, 62(2), 487-498.

Mozersky, J., Sankar, P., Harkins, K., Hachey, S. und Karlawish, J. (2018): Comprehension of an elevated amyloid positron emission tomography biomarker result by cognitively normal older adults. Jama Neurology, 75(1), 44-50.

Mozersky, J., Hartz, S., Linnenbringer, E., Levin, L., Streitz, M., Stock, K., Moulder, K. und Morris, J. C. (2021): Communicating 5-Year Risk of Alzheimer's Disease Dementia: Development and Evaluation of Materials that Incorporate Multiple Genetic and Biomarker Research Results. Journal of Alzheimer's disease: JAD, 79(2), 559–572.

Jessen, F., Amariglio, R. E., van Boxtel, M., Breteler, M., Ceccaldi, M., Chételat, G., Dubois, B., Dufouil, C., Ellis, K. A., van der Flier, W. M., Glodzik, L., van Harten, A. C., de Leon, M. J., McHugh, P., Mielke, M. M., Molinuevo, J. L., Mosconi, L., Osorio, R. S., Perrotin, A., Petersen, R. C., … und Subjective Cognitive Decline Initiative (SCD-I) Working Group (2014): A conceptual framework for research on subjective cognitive decline in preclinical Alzheimer's disease. Alzheimer's & dementia : the journal of the Alzheimer's Association, 10(6), 844–852.

Samerski, S., & Henkel, A. (2015). Responsibilisierende Entscheidungen. Strategien und Paradoxien des sozialen Umgangs mit probabilistischen Risiken am Beispiel der Medizin. Berliner Journal für Soziologie, 25(1), 83-110.

Samerski, S. (2015). The decision trap. Genetic Education and its Social Consequences. Exeter, Devon: Imprint Academic.

Shaw, L. M., Arias, J., Blennow, K., Galasko, D., Molinuevo, J. L., Salloway, S., Schindler, S., Carrillo, M. C., Hendrix, J. A., Ross, A., Illes, J., Ramus, C. und Fifer, S. (2018): Appropriate use criteria for lumbar puncture and cerebrospinal fluid testing in the diagnosis of Alzheimer's disease. Alzheimer's & dementia : the journal of the Alzheimer's Association, 14(11), 1505–1521.

Simonsen, A. H., Herukka, S. K., Andreasen, N., Baldeiras, I., Bjerke, M., Blennow, K. und Waldemar, G. (2017): Recommendations for CSF AD biomarkers in the diagnostic evaluation of dementia. Alzheimer's & Dementia, 13(3), 274-284.

Tijms, B. M., Bertens, D., Slot, R. E., Gouw, A. A., Teunissen, C. E., Scheltens, P., van der Flier, W. M. und Visser, P. J. (2017): Low normal cerebrospinal fluid Aβ42 levels predict clinical progression in nondemented subjects. Annals of neurology, 81(5), 749–753.

van Maurik, I. S., Slot, R., Verfaillie, S., Zwan, M. D., Bouwman, F. H., Prins, N. D., Teunissen, C. E., Scheltens, P., Barkhof, F., Wattjes, M. P., Molinuevo, J. L., Rami, L., Wolfsgruber, S., Peters, O., Jessen, F., Berkhof, J., van der Flier, W. M. und Alzheimer’s Disease Neuroimaging Initiative (2019): Personalized risk for clinical progression in cognitively normal subjects-the ABIDE project. Alzheimer's research & therapy, 11(1), 33.

von Unger, H. (2014). Forschungsethik in der qualitativen Forschung: Grundsätze, Debatten und offene Fragen. In: Forschungsethik in der qualitativen Forschung. Springer VS, Wiesbaden. S. 15-39.

Wolfsgruber, S., Polcher, A., Koppara, A., Kleineidam, L., Frölich, L., Peters, O., Hüll, M., Rüther, E., Wiltfang, J., Maier, W., Kornhuber, J., Lewczuk, P., Jessen, F. und Wagner, M. (2017): Cerebrospinal Fluid Biomarkers and Clinical Progression in Patients with Subjective Cognitive Decline and Mild Cognitive Impairment. Journal of Alzheimer's disease : JAD, 58(3), 939–950.

Literaturverzeichnis Testbatterie:

MMSE

F. Folstein, S. E. Folstein, P. R. McHugh: Mini-Mental State (a practical method for grading the state of patients for the clinician). In: Journal of Psychiatric Research. 12, 1975.

Objective and Subjective Numeracy Scale

García-Retamero, R., & Galesic, M. (Eds.). (2013). Transparent communication of health risks: Overcoming cultural differences. New York: Springer.

Following: Making numbers matter: present and future research in risk communication.Fagerlin A, Ubel PA, Smith DM, Zikmund-Fisher BJ. Am J Health Behav. 2007

FLZM

Henrich, G. und Herschbach, P.: Questions on Life Satisfaction (FLZM) - A short questionnaire for assessing subjective quality of life. European Journal of Psycholo-gical Assessment, 2000

BRS

Smith, B. W., Dalen, J., Wiggins, K., Tooley, E., Christopher, P., & Bernard, J. (2008). The brief resilience scale: assessing the ability to bounce back. International journal of behavioral medicine, 15(3), 194-200.

BFI-10

Rammstedt, B., Kemper, C. J., Klein, M. C., Beierlein, C., & Kovaleva, A. (2014). Big Five Inventory (BFI-10).

HADS

Zigmond AS, Snaith RP. The hospital anxiety and depression scale. Acta Psychiatr Scand. 1983;67:361–70.

UCLA Loneliness

Hughes ME, Waite LJ, Hawkley LC, Cacioppo JT. A Short Scale for Measuring Loneliness in Large Surveys: Results From Two Population-Based Studies. Res Aging. 2004;26(6):655-672. doi: 10.1177/0164027504268574. PMID: 18504506; PMCID: PMC2394670.

SCD questionnaire

The SCD questionnaire designed to capture the SCD-plus criteria which are features of SCD that in the current state of knowledge are associated with increased likelihood of underlying AD pathology.

Following: Jessen F, Spottke A, Boecker H, et al. Design and first baseline data of the DZNE multicenter observational study on predementia Alzheimer's disease (DELCODE). Alzheimers Res Ther. 2018;10(1):15. Published 2018 Feb 7. doi:10.1186/s13195-017-0314-2

Jessen F, Amariglio RE, van Boxtel M, Breteler M, Ceccaldi M, Chételat G, et al. A conceptual framework for research on subjective cognitive decline in preclinical Alzheimer's disease. Alzheimers Dement. 2014;10:844–52.
