# Supplementary material for: Dementia risk estimation in persons at risk and the predictive turn in Alzheimer’s disease—The PreTAD project: Study protocol with an ethical, clinical, linguistic, and legal approach
Source: PLoS One. 2025 Jul 16;20(7):e0319868. doi: 10.1371/journal.pone.0319868 (PMC12266435; doi:10.1371/journal.pone.0319868)
Supplement: S3 File — (PDF) [file pone.0319868.s003.pdf]

Study protocol project: "Prediction of Alzheimer's disease: Ethical, clinical, linguistic and legal aspects of the paradigm shift of predictive medicine" (PreTAD)

Summary of the project

Background: In medicine today, a paradigm shift is emerging in which diagnostics and therapy based on disease symptoms are being supplemented or even replaced by early detection, risk prediction and prevention. With regard to Alzheimer's disease (AD), diagnostic methods for early detection and risk prediction are constantly evolving, with increasing diagnostic accuracy. Blood-based biomarkers could clinically applied but comparatively invasive and expensive diagnostics (e.g. CSF and PET) for the detection of Alzheimer's pathology. The resulting higher availability may lead to increased use risk prediction in this context, e.g. including patients with subjective cognitive disorder (SCD) or symptom-free individuals. The PreTAD project to provide data on people's different perspectives on the prediction of Alzheimer's dementia and the impact of predictive medicine on individuals and society from clinical, ethical, legal and linguistic perspectives. The PreTAD project is being carried out in collaboration at three locations (Cologne/Bonn, Geneva and Barcelona).

Research question: The ethical debate on risk prediction at the individual and societal level encompasses medical, cultural, legal and philosophical aspects. Emerging blood-based biomarkers could greatly facilitate access to Alzheimer's risk prediction. This could increase the desire and pressure to perform Alzheimer's biomarker diagnostics not only in cognitively impaired but also in preclinical and asymptomatic individuals. As a tri-national collaborative project, the PreTAD study aims to help establish a framework for good clinical practice in the prediction of Alzheimer's disease in pre-symptomatic and early (subjective) symptomatic stages.

Method: We intend to concentrate recruitment on a total of five different groups of test subjects:

(a) 8-10 cognitively healthy individuals with no family history or personal experience of Alzheimer's dementia (conducted by the German sites only), (b) 150 healthy participants (50 participants per site) with a potentially higher risk of developing Alzheimer's dementia due to a family history of Alzheimer's disease in first-degree relatives or due to evidence of an APOE4 allele, (c) 150 participants(50 participants per site) with previous SCD diagnosis ≤ 2 years and > 4 weeks, (d) 90 participants (30 participants per site) with new SCD diagnosis (≤ 4 weeks), and (e) N≈1800 individuals from the general population (≈1000 in Germany, ≈500 in Spain, ≈300 in Switzerland) (conducted by the German sites only). To achieve a comprehensive approach, the interdisciplinary collaboration combines a quantitative assessment, qualitative (narrative) interviews (groups a-d, German sites only) and empirically supported theoretical approaches to ethical, social and legal analysis.

Study participants will be surveyed using a quantitative test battery with partially newly developed questionnaires, as well as hypothetical scenarios to reveal Alzheimer's biomarker findings and dementia risk. Groups a-d will receive the entire questionnaire battery, while group e will participate in an online cross-sectional survey and receive a shortened version of the questionnaire battery to increase user-friendliness and achieve a representative sample size in each country. The longitudinal study (group b-d) will be conducted by specialized centers for memory disorders in Cologne, Geneva and Barcelona over a period 12 months.

As part of the qualitative approach of PreTAD, which is only conducted at the German site, a total of 40 narrative interviews with N=40 participants will be conducted at the beginning of the study with individuals from groups a-d (n=8-10 per group). The study participants be offered an optional follow-up interview 1 year after the baseline interview. If participants from groups c + d are accompanied by relatives to the interview appointment, they will also be invited to participate in the interviews.

In an initial pilot study at the German site, the feasibility of the comprehensive questionnaire battery for the quantitative approach was tested with N=20 participants (with or without diagnosed SCD). For the qualitative approach, the newly developed interview guidelines were with n=8 participants from groups a-d. Shortened questionnaires for group e were tested with N=24 participants.

Results and practical implications: The data we provide as part of the clinical-empirical approach serve to clarify and explore the perspectives, needs, and preferences of cognitively healthy individuals at increased risk for developing Alzheimer's dementia and individuals in preclinical stages with respect to screening for early Alzheimer's biomarker diagnostics and prediction of Alzheimer's dementia. Our assessment provides information on the general numerical understanding of risk and expectations for risk prediction. It also focuses on psychological distress during the process of risk prediction, subjective well-being, quality of life (QoL), identity and self-perception, as well as readiness for lifestyle changes and preventive measures.

Responsibilities

The principal investigator of this project is Prof. Dr. Christiane Woopen, holder of the Heinrich Hertz Professorship for Ethics of Life, TRA4 at the University of Bonn, Germany. At the German sites, the study is being conducted at the Center for Life Ethics at the University of Bonn and the Center for Memory Disorders at the Clinic and Polyclinic for Psychiatry and Psychotherapy at the University Hospital of Cologne. In Cologne, Dr. Ayda Rostamzadeh is responsible for conducting the quantitative survey of groups b-d. The Center for Memory Disorders at the University Hospital of Cologne is also responsible for recruiting the subject groups b-d. In Barcelona, Prof. Dr. Boada, founder of the Ace Alzheimer Center Barcelona, Universitat Internacional de Catalunya, is for conducting the quantitative survey of the b-d groups. In Geneva, Prof. Dr. Frisoni, Head of the Department of Rehabilitation and Geriatrics of the Geneva Memory Center at the University Hospital of Geneva, is responsible for conducting the surveys of groups b-d at this site. The Center for Life Ethics at the University of Bonn is responsible for the recruitment of participants for both the quantitative and qualitative survey of group a (German site only) and group e (online survey, all sites). The project is funded by the ERA-NET NEURON (Network of European Funding of Neuroscience Research) and the German Federal Ministry of Education and Research (BMBF).

This study project was entered in the German study register after approval by the coordinating ethics committee of the University of Bonn (DRKS ID: DRKS00029035).

Scientific background

The social impact of a paradigm shift towards predictive medicine has so far been the subject of comparatively little research. The lack of treatment methods for cognitive impairment in the course of Alzheimer's disease (AD) is leading to a considerable expansion of predictive methods with the aim of ever earlier prediction, which is why this area is a very suitable example for the study of the "predictive turn" medicine on

can be seen on different levels. At the same time, neurodegenerative diseases that cause dementia, such as AD, have become a major public health challenge. Nevertheless, studies on the effects of early dementia prediction in the field of AD research are still rare and mostly measure the effects at the individual level. Overall, however, the urgency of a "review of the ethical framework" (Angehrn et al., 2019) is emphasized.

AD, as the pathophysiological cause of Alzheimer's dementia, begins decades before symptoms appear. Early detection of AD in preclinical and prodromal stages is possible long before the functional limitations of dementia set in (Jack et al., 2018), with the preclinical phase of AD being described by the stage of subjective cognitive impairment. Within this, a persistent subjective observation of cognitive impairment compared to the individual's baseline level is noted. While SCD initially describes a non-specific and heterogeneous syndrome that may not be associated with AD in older individuals, recent research shows that individuals with SCD and biomarker evidence AD have an increased risk of subsequent cognitive decline (Wolfsgruber et al, 2017; Tijms et al, 2017; Van Maurik et al, 2019; Ebenau et al, 2020). The available clinical study data indicate that there are numerous hurdles and uncertainties in the communication of AD biomarker results in cognitively healthy individuals. These result from the possibly unclear conceptual understanding of predictive diagnostics and the assessment of risk probabilities in this group of people, in contrast to definitive diagnoses (Mozersky et al., 2018). In particular, when communicating a risk assessment, there is a danger that a risk of illness will be confused with a diagnosis of illness (Milne et al., 2018). Furthermore, interviews with cognitively healthy subjects who received amyloid positron emission tomography (PET) imaging as part of the SOKRATES (Study of Knowledge and Reactions to Amyloid Testing) study showed that there are concerns about stigmatization due to pathological findings and that the test results are significant for the identity, self-determination and social interactions of the respondents (Largent et al., 2020).

While predictive methods are becoming safer, more affordable and more easily accessible, new questions are arising that touch on a wide range of disciplinary domains. Ethically and anthropologically, for example, changes in people's self-perception, the potential for discrimination and (self-)stigmatization must be considered, as well as influences on self-determination and social relationships; sociologically and ethically, it is a matter of understanding social contracts and principles in healthcare, including solidarity; socio-economically and legally, the changes in healthcare systems and their financing, as well as regulatory instruments for ensuring quality and freedom in medical treatment, must be reassessed. This also includes medical indications, information and consent as well as standards of care and liability.

In particular, when communicating a risk assessment as part of a screening examination, there is a danger that a disease risk will be confused with a disease diagnosis. It has been shown that in cognitively healthy individuals, the conceptual understanding of predictive diagnostics and risk probabilities, as opposed to definitive diagnoses, presents a challenge (Mozersky et al., 2018; Milne et al. 2018). The consideration of language and communication in the context of clinical AD research is currently limited to the creation of counseling and information materials (Samerski/ Henkel (2015); Samerski (2015)). Only rarely is the variety of (communicative) possibilities of individual risk perception and processing or even

takes into account the reflection of habitualized communicative practices and linguistic patterns in the field predictive medicine and risk communication (Lorke et al. 2021).

Looking at the needs in clinical practice, there are first publications on handouts for cognitively healthy study subjects participating in AD screening tests (Mozersky et al., 2021). Although recently published recommendations on CSF- and PET-based AD biomarker testing provide a framework for AD diagnostics from the symptomatic stage of AD, recommendations on counseling and reporting of findings for cognitively healthy individuals and individuals with SCD are lacking (Harkins et al., 2015; Simonsen et al., 2017; Johnson et al., 2013; Shaw et al., 2018).

Especially due to the heavy burden associated with AD and the strong presence of the disease in the media discourse, there is increasing hope for an effective treatment in the future. The emerging possibilities of determining biomarkers from the blood could the desire and demand for AD biomarker testing in asymptomatic individuals and individuals with SCD. This increases the urgency of the above-mentioned research questions and the interdisciplinary and multi-perspective approach of this project.

Project goals

The overall aim of PreTAD is to identify individual needs and different perspectives of people in relation to the prediction of AD and to discuss the implications of the paradigm shift in medicine on an individual, linguistic, legal and societal level.

In order to clarify the individual and societal needs with regard to healthcare systems and their services, the different perspectives on prediction and the specific decision parameters (for or against prediction) that play a role, the study will include people with SCD and people with a family history of AD or APOE4 allele carriers as well as healthy people (general population) in the individual interviews. In addition, the discursive, public-media perspective is examined in order to integrate social opinion into the analysis.

By investigating the effects of predictive medicine on the individual, the respective society and the healthcare system using the example of AD, the ultimate aim is to develop a framework and guidelines for the presymptomatic prediction of Alzheimer's dementia, which can be helpful for clinical practice. In addition, the findings from PreTAD will be reflected in scientific publications (open access) during the course of the project and made publicly available so that they can contribute to the scientific discussion in the relevant specialist areas.

Target values

In a mixed-methods research design, PreTAD combines qualitative and quantitative data to analyze the collected data.

In the ethical sub-project of PreTAD, a mixed-methods approach is used, which is based on the combination of the results of the statistical survey analysis (quantitative data) and a structured thematic analysis of the qualitative data, which leads to ethical assessments and typical differentiations. The interview data is evaluated using the analysis tool 'MAXQDA'.

The empirical data collected as part of the clinical sub-project of PreTAD enable cross-national comparability. The statistical methods used in this sub-project include parametric approaches for group comparisons (e.g. T-statistics, analysis of variance) and regression analyses for association testing.

For the linguistic sub-project of PreTAD, the transcribed interviews are also analyzed with the analysis tool 'MAXQDA' due to the close connection with the ethical sub-project. In this sub-project, linguistic conversation analysis is used to analyze the interviews. This makes it possible to focus on the use of language and the communicative practices employed.

In the analysis of the legal sub-project of PreTAD, classical methods of system analysis, legal interpretation, the interpretation of regulations, the creation of regulatory guidelines and specifications, legal comparison, in particular taking into account the systems of Switzerland, Spain and Germany, and the identification of sticking points with a need for regulation or political action are applied.

Study population

Overview:

Participants	• Group a: 8-10 cognitively healthy people with no family history or personal experience of Alzheimer's dementia (Germany only)<br>• Group b: 150 cognitively healthy individuals with a positive family history or APOE4 status (n=50 per site)<br>• Group c: 150 people with subjective cognitive impairment (SCD) (previous SCD diagnosis > 4 weeks and ≤ 2 years) (n=50 per site)<br>• Group d: 90 people with SCD (new SCD diagnosis≤ 4 weeks) (n= 30 per site)<br>• Group e: 1,800 people from the general population (in Germany ≈ 1,000, Spain≈ 500, Switzerland≈ 300)

Inclusion and exclusion criteria:

General inclusion criteria (survey and interviews)	• At least 18 years old<br>• Sufficient command of the national language of the respective study location so that informed consent can be given and the completion of the questionnaires can be guaranteed<br>• Interviews only: sufficient command of German so that the qualitative interviews can be conducted in German.<br>can be
General exclusion criteria (survey and interviews)	• Insufficient knowledge of the local language<br>• Illiteracy<br>• Persons who are not capable of giving consent or have not given written consent

In addition to the general inclusion and exclusion criteria, the following group-specific criteria apply:

<u>*Group a*</u> (cognitively healthy individuals with no family history or personal experience of Alzheimer's dementia (Germany only)):

Additional Inclusion criteria	None
Additional exclusion criteria	• Subjective or objective cognitive impairment<br>• Moderate to severe depressive disorder; Hospital Anxiety and Depression Scale (HADS) score > 10 points<br>• Close contact with a person with Alzheimer's disease<br>• (Previous) Visit to an Alzheimer's prevention center or comparable facility<br>• Persons with known ApoE-e3/e4 or ApoE-e4/e4 gene carrier status

<u>*Group b*</u> (participants with first-degree relatives who have been diagnosed with Alzheimer's disease or who are carriers of the ApoE4 gene):

Additional inclusion criteria	• Knowledge of a medical diagnosis of Alzheimer's disease in at least one first-degree relative (mother, father, sibling) or<br>• Participants: with a ApoE-e3/e4- or ApoE-e4/e4- Gene carrier status
Additional exclusion criteria	• Subjective or objective cognitive impairment<br>• Moderate to severe depressive disorder; Hospital Anxiety and Depression Scale (HADS) score > 10 points

<u>*Group c-d*</u> (participants with an SCD diagnosis> 4 weeks and≤ 2 years (group c) and≤ 4 weeks (group d)):

Additional inclusion criteria	• Clinical criteria for the diagnosis of SCD (according to the criteria of Jessen et al. 2014)> 4 weeks and≤ 2 years (group c) or≤ 4 weeks (group d)<br>• A subjective and persistent (non-acute) deterioration in cognitive performance compared to the original baseline level that is not due to an acute event<br>• Neuropsychological test battery for mild cognitive impairment (MCI) or prodromal Alzheimer's disease shows a score within the age-, sex-, and gender-specific range.<br>and education-adjusted norm group
Additional exclusion criteria	• MCI, prodromal AD or dementia<br>• Impairments can be explained by a psychiatric* or neurological illness (except Alzheimer's), somatic illness, medication or substance abuse<br>• Moderate to severe depressive disorder; HADS score> 10 points

	*Mild subsyndromal depressive symptoms or anxiety symptoms are not considered exclusion criteria.

Group e (people from the general population):

Additional Inclusion /Exclusion criteria	None

The same inclusion and exclusion criteria apply to the participants in the pilot studies.

Recruitment

Recruitment for the questionnaire study is planned as follows:

Group a (cognitively healthy general population with no family history or personal experience of Alzheimer's dementia, German site only)

The participants in group a are recruited via (social) media. In addition, the snowball system strategy (participants recommend interested acquaintances or friends) can also be used for recruitment. The survey and interview will be conducted in person at one of the German study locations or via Zoom. Before the survey and interview, participants will be informed about the purpose of the study and how the data will be used and stored. Participants must then give their written consent to participate in the study.

Group b (participants with first-degree relatives who have been diagnosed with Alzheimer's or who are ApoE4 gene carriers)

Subjects from group b are recruited via the Cologne Alzheimer's Prevention Register. Contact is made via a circular email with a reference to the PreTAD study. Contact information for participation in the study is attached to this email.

Group c and d (participants with SCD diagnosis > 4 weeks and ≤ 2 years (group c) and ≤ 4 weeks (group d))

Participants in groups c and d in Germany are recruited via the Center for Memory Disorders at the University Hospital of Cologne by personal contact.

Group e (people from the general population):

The participants in group e are recruited via an external service provider that specializes in conducting large-scale surveys for the academic sector.

The study plan for all study arms is shown below:

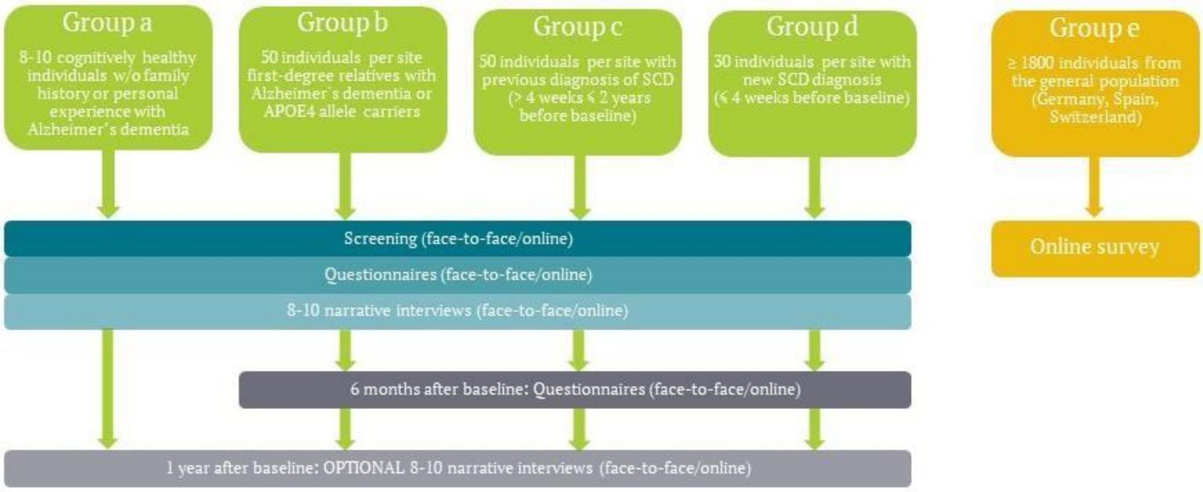

Figure 1 PreTAD study and recruitment plan

Methodology and implementation

This project is a tricentric study project. The project is being carried out in Germany (Bonn, Cologne), Switzerland (Geneva) and Spain (Barcelona). The interview study will only be conducted in Germany.

PreTAD is aimed at five participant groups in order to take into account the differentiated view of the healthy population with different types of contact with memory clinics and Alzheimer's dementia (groups a-d) and the general population (group e). All potential participants will receive information sheets about the study, the objectives of the study, the exact study procedure, information on the voluntary nature and pseudonymization of the data, information on new findings, information on participant insurance (groups a-d) and the contact details of the study center. If they then decide to participate in the study, participants will receive a declaration of consent in which they agree to the study and the data protection regulations. The declaration of consent must be submitted at the beginning of the respective study.

The information and declarations of consent for the online survey of the general population (group e) are displayed on the first page of the survey tool and can be confirmed by clicking on them.

The project is divided into two study parts: In the questionnaire survey, topics such as lifestyle, knowledge about prediction and risk as well as questions about needs and perspectives towards Alzheimer's dementia prediction/new testing procedures (considerations/parameters) will be asked. In addition, questions on the assessment of hypothetical scenarios of risk communication on Alzheimer's dementia are included. In the interview study, narrative interviews are conducted to collect additional aspects and facets of individual perspectives on disease prediction in cognitively healthy individuals with different previous experiences with the topic (group a-d). Ten participants per group (a-d) will be included in the interview study.

German sites only: Participants taking part in the interview study (groups b-d) will receive the information and informed consent forms from the study doctor if they or the study doctor are not participating in the study.

respective family members attend their regular appointment at the Cologne Alzheimer's Prevention Center. If potential study participants from group b indicate that a face-to-face appointment is not an option, the research team offers the option of conducting the interview via Zoom, as with group a, which receives all the necessary documents by email.

Questionnaire study / online survey:

In the quantitative survey (questionnaire study, groups a-d), attitudes and needs as well as reservations of the subjects towards new predictive possibilities will be investigated using questionnaires using Alzheimer's dementia as an example. We will use validated questionnaires that are widely used in clinical psychology and are part of the regular assessment of patients in memory clinics. In addition, we will use newly developed questionnaires that reflect the aims of the study with regard to individual attitudes and perspectives on the prediction of Alzheimer's dementia.

Participants in groups b-d will be made aware of the opportunity to participate in the study on site at the Center for Memory Disorders (Clinic and Polyclinic for Psychiatry and Psychotherapy at Cologne University Hospital) or via the Alzheimer's Prevention Register. The time required for the participants is a total of 45 minutes for the screening examination and approx. 90 minutes for the baseline and final examination (6-month follow-up).

The shortened version of the questionnaire battery for the general population (group e) has a duration of approx. 15 minutes. The following instruments, some of which have been validated but also newly developed, are to be used in PreTAD:

Questionnaire	***Groups a-d***	***Group e***
Pre-existing		
Mini Mental State Examination (MMSE) (Screening instrument)	X	-
Hospital Anxiety and Depression Scale (HADS) (Screening-instrument)	X	-
SCD questionnaire (screening instrument)	X	-
Lifestyle questionnaire	X	Only one item
Life Satisfaction Questionnaire (FLZM)	X	-
Subjective understanding of numbers	X	-
Objective understanding of numbers	X	-
Loneliness (UCLA)	X	-
Brief Resilience Scale (BRS)	X	X
Big Five inventory	X	X
Newly developed for PreTAD		
Demographic data	X	Abridged version
Previous experience with dementia	X	Abridged version
Needs and decision parameters in Reference to Alzheimer's screening and new test methods	X	Abridged version
Subjective risk perception	X	Abridged version
Evaluation of hypothetical scenarios of AD biomarkers: Reporting of findings and risk prediction	X	Abridged version (without graphics)

Table 1: Overview of the test battery for all groups.

The quantitative data from the questionnaire study is recorded and stored in REDCap (groups a- d). REDCap is a browser-based, metadata-driven EDC software. The data is not stored on

stored on a central server, but on the server of the institution that uses the software (here: University of Cologne). All project data is stored and hosted by the local institution and is not forwarded to other institutions or organizations. As no international servers outside the EU are involved, REDCap can be used to meet the requirements of the EU General Data Protection Regulation (GDPR). The rights to add, change or delete data can be assigned individually by the administrator.

The quantitative data of Group e is processed using the GDPR-compliant guidelines of the relevant external service provider.

Interview study:

To evaluate the interviews at the German study location, they are recorded with an audio device with the consent of the participants and transcribed by a service provider for further data analysis. The interviews are analyzed using a qualitative content analysis according to Kuckartz (2016).

The expected end of data collection is November 2023. The first interviews will be transcribed and successively analyzed during the interview study period.

Preliminary study:

A preliminary study was conducted at the Cologne site between January and February 2023 to test the newly developed questionnaires with n=10 healthy subjects and n=10 subjects with SCD. The questionnaires were checked for their feasibility and comprehensibility. The quantitative pilot surveys were conducted on paper and lasted around 60 minutes. The interview guide for the qualitative study was also tested for feasibility as part of the preliminary study with n=2 healthy people and n=6 test subjects with SCD and took approximately 60 minutes.

Interim evaluation and termination criteria

Participants are given the opportunity to discontinue participation in the study at any time without giving reasons or to refuse to answer individual questions. The participants will be informed that this will not them at any disadvantage. If, in the course of the study, a subject no longer meets the inclusion criteria, the participation of the subject and their relatives in the study will be terminated.

A premature end to the study only comes into effect if the fundamental conditions that led to the approval of the study are revoked by the ethics committee or the BMBF

Risk-benefit assessment

As this is a non-interventional study, no complications or risks are expected.

The rules for interviewing adults must always be observed (ADM 2021). From a research ethics perspective, the design of this study does not give rise to any concerns regarding emotional distress caused by participation in this study.

There is no direct benefit for the participants of this study, but the reflection on the prediction of diseases in general and the prediction of Alzheimer's disease in particular can be of great benefit.

Dementia in particular could evoked by participation, and raising awareness of this topic could be a positive consequence of participation. On the other hand, participation could also trigger negative consequences such as fears or insecurities in dealing with this topic with regard to the participants' own health. In principle, the implementation of quantitative and qualitative study designs requires careful and continuous reflection on the part of the researchers in order to with the principle of non-harming participants. This concerns the aspects of integrity of the researchers, non-deception of the study participants, informed consent and voluntary participationavoidance of harm before, during and after the study as well as confidentiality and pseudonymization of the results (von Unger 2014; Hopf 2016).

It is ensured that the subjects of this study have been fully informed in advance and thus enabled to give informed consent. In addition, participation is voluntary at all times and subjects are informed that they have the right to withdraw their consent at any time without this having any negative consequences for them. The confidentiality of the results is also guaranteed; in the event of publication, these are to be published in anonymized form in accordance with the data protection guidelines.

Neither in the quantitative survey nor in the qualitative survey can it be ruled out that sensitive or stressful topics in connection with medical predictive measures or Alzheimer's dementia topics will be discussed. Concrete measures to minimize this risk are repeated indications that the respondents can decide completely independently what, how much and about what they say. Burdens for the respondents or possible risks before, during and after data collection can be considered low overall in this study.

Online survey/questionnaire study:

As the questions are designed to be varied and the questionnaires also be completed by respondents at a later date in groups e and a (online survey), i.e. at home or at a location of their choice, the duration of the study is not expected to be burdensome. Completion of the questionnaires for groups b-d will be supervised by trained staff. Emotional distress is unlikely, but some questions may be cognitively demanding for respondents.

Interview study:

Emotional stress is considered unlikely. Nevertheless, individual everyday situations in connection with the prediction of Alzheimer's dementia, which are perceived more sensitively through participation in the study, could represent a psychological burden. The planned duration of the interview study of 90 minutes could also be perceived as time-consuming by individual participants. If consent is given, the participants will take part in this interview study.

Biometrics

As PreTAD is primarily an exploratory study, no sample size calculation will be performed for the endpoints with expected effect sizes in groups a-d. The planned analyses focus in particular on the differences between the genders of the subjects and the different study populations as well as on the transnational comparability of the study countries. Parametric approaches of group comparison (e.g. T-statistics, analysis of variance) and regression analyses for association testing will be applied. Representativeness is sought for group e and a power calculation is carried out.

The qualitative interview data collected at German locations is evaluated using the MAXQDA analysis tool and subjected to a structured thematic analysis according to Kuckartz (2017).

Data management and data protection

Interview study:

If contact restrictions are imposed at a later date for safety reasons (e.g. Covid pandemic), the interview study will be conducted online with all groups (a-d). The interview study will be conducted online using the video conferencing tool "Zoom" (https://rrzk.uni- koeln.de/support-information/information-zu-tools-fuer-kollaboratives- arbeiten/zoom-datenschutz-und-nutzungsvorgaben-fuer-hosts-moderatorinnen), which is used by the University of Cologne.

The reason for this is that the service provider well-known among the German population, easy to use and very efficient. All participants receive a link to participate in the interview study with the corresponding passwords. For the documentation and evaluation of the interviews, the audio is recorded using an Olympus audio system recording device so that the data is stored locally on the servers of the University of Cologne for evaluation exclusively independently of the service provider "Zoom". At the beginning of the study, all interviewees complete a preliminary questionnaire on their socio-demographic data and send it back to the research team by post together with the declaration of consent

Interview study and online survey:

All personal data will be stored in tabular form exclusively for the purpose of contacting and processing the study. Participants will be assigned a study ID. This pseudonymization list is stored separately from the collected data on the local server of the University of Cologne for the duration of the data analysis. Only the principal investigator, the study coordinator and the scientific staff of the PreTAD research team have access to the pseudonymization list. Only the study IDs are used in the data material itself. Respondents are informed that their data will only be processed in pseudonymized form and that the researchers involved are obliged to maintain confidentiality regarding personal information. The data collected will only be used in pseudonymized form for scientific research purposes and will only be passed on to the countries involved in the project (Switzerland and Spain) in anonymized form. The pure audio tracks of the interview study are passed on to the service provider for transcription. The interviewees will be informed about this transfer. The data will not be passed on to other third parties. Only anonymized data will be used for scientific publications. Respondents can end their participation in the study at any time without consequences and withdraw their consent to the use of their data. In this case, all data collected from this person will be deleted. The applicable data protection regulations in accordance with the GDPR are complied with. The right to be "forgotten" by deletion of the data contained therein is guaranteed.

Bibliography

Angehrn, Z., Nordon, C. and Turner, A. (2019): Ethical and social implications of using predictive modeling for Alzheimer's disease prevention: a systematic literature review protocol. BMJ Open. 9, e026468.

Corbin, J. M. and Strauss, A. (1990). Grounded theory research: Procedures, canons, and evaluative criteria. Qualitative sociology, 13(1), 3-21.

Ebenau, J. L., Timmers, T., Wesselman, L., Verberk, I., Verfaillie, S., Slot, R., van Harten, A. C., Teunissen, C. E., Barkhof, F., van den Bosch, K. A., van Leeuwenstijn, M., Tomassen, J., Braber, A. D., Visser, P. J., Prins, N. D., Sikkes, S., Scheltens, P., van Berckel, B. and van der Flier, W. M. (2020): ATN classification and clinical progression in subjective cognitive decline: The SCIENCe project. Neurology, 95(1), e46-e58.

Flick, U. (2019): Qualitative social research. An introduction. Original edition, 9th edition. Reinbek bei Hamburg: rowohlts Enzyklopädie im Rowohlt Taschenbuch Verlag (Rororo Rowohlts Enzyklopädie, 55694).

Harkins, K., Sankar, P., Sperling, R., Grill, J. D., Green, R. C., Johnson, K. A. and Karlawish, J. (2015): Development of a process to disclose amyloid imaging results to cognitively normal older adult research participants. Alzheimer's research & therapy, 7(1), 1-9.

Hopf, C. (2016): Research ethics and qualitative research. In: Schriften zu Methodologie und Methoden qualitativer Sozialforschung. Springer VS, Wiesbaden. S. 195-205.

Jack Jr, C. R., Bennett, D. A., Blennow, K., Carrillo, M. C., Dunn, B., Haeberlein, S. B. and Silverberg, N. (2018): NIA-AA research framework: toward a biological definition of Alzheimer's disease. Alzheimer's & Dementia, 14(4), 535-562.

Johnson, K. A., Sperling, R. A., Gidicsin, C. M., Carmasin, J. S., Maye, J. E., Coleman, R. E. and AV45-A11 study group. (2013): Florbetapir (F18-AV-45) PET to assess amyloid burden in Alzheimer's disease dementia, mild cognitive impairment, and normal aging. Alzheimer's & Dementia, 9(5), 72-83.

Kuckartz, U. (2017): Computer-aided content analysis. In: Lothar Mikos and Claudia Wegener (eds.): Qualitative Media Research. A handbook. 2nd, completely revised and expanded edition. Constance, Munich: UVK Verlagsgesellschaft mbH; UVK Lucius (utb Medien- und Kommunikationswissenschaft, Pädagogik, Psychologie, Soziologie, 8314), pp. 503-515.

Largent, E. A., Harkins, K., van Dyck, C. H., Hachey, S., Sankar, P., and Karlawish, J. (2020): Cognitively unimpaired adults' reactions to disclosure of amyloid PET scan results. PLoS One, 15(2), e0229137.

Lorke, M., Schwegler, C., & Jünger, S. (2021). Re-claiming the power of definition-The value of reflexivity in research on mental health at risk. In Qualitative Research Methods in Mental Health (pp. 135-165). Springer, Cham.

Milne, R., Bunnik, E., Diaz, A., Richard, E., Badger, S., Gove, D. and Brayne, C. (2018): Perspectives on communicating biomarker-based assessments of Alzheimer's disease to cognitively healthy individuals. Journal of Alzheimer's Disease, 62(2), 487-498.

Mozersky, J., Sankar, P., Harkins, K., Hachey, S. and Karlawish, J. (2018): Comprehension of an elevated amyloid positron emission tomography biomarker result by cognitively normal older adults. Jama Neurology, 75(1), 44-50.

Mozersky, J., Hartz, S., Linnenbringer, E., Levin, L., Streitz, M., Stock, K., Moulder, K. and Morris, J. C. (2021): Communicating 5-Year Risk of Alzheimer's Disease Dementia: Development and Evaluation of Materials that Incorporate Multiple Genetic and Biomarker Research Results. Journal of Alzheimer's disease: JAD, 79(2), 559-572.

Jessen, F., Amariglio, R. E., van Boxtel, M., Breteler, M., Ceccaldi, M., Chételat, G., Dubois, B., Dufouil, C., Ellis, K. A., van der Flier, W. M., Glodzik, L., van Harten, A. C., de Leon, M. J., McHugh, P., Mielke, M. M., Molinuevo, J. L., Mosconi, L., Osorio, R. S., Perrotin, A., Petersen, R. C., ... and Subjective Cognitive Decline Initiative (SCD-I) Working Group (2014): A conceptual framework for research on subjective cognitive decline in preclinical Alzheimer's disease. Alzheimer's & dementia : the journal of the Alzheimer's Association, 10(6), 844-852.

Samerski, S., & Henkel, A. (2015). Responsibilizing decisions. Strategies and paradoxes of the social handling of probabilistic risks using the example of medicine. Berlin Journal of Sociology, 25(1), 83-110.

Samerski, S. (2015). The decision trap. Genetic Education and its Social Consequences. Exeter, Devon: Imprint Academic.

Shaw, L. M., Arias, J., Blennow, K., Galasko, D., Molinuevo, J. L., Salloway, S., Schindler, S., Carrillo, M. C., Hendrix, J. A., Ross, A., Illes, J., Ramus, C. and Fifer, S. (2018): Appropriate use criteria for lumbar puncture and cerebrospinal fluid testing in the diagnosis of Alzheimer's disease. Alzheimer's & dementia : the journal of the Alzheimer's Association, 14(11), 1505-1521.

Simonsen, A. H., Herukka, S. K., Andreasen, N., Baldeiras, I., Bjerke, M., Blennow, K. and Waldemar, G. (2017): Recommendations for CSF AD biomarkers in the diagnostic evaluation of dementia. Alzheimer's & Dementia, 13(3), 274-284.

Tijms, B. M., Bertens, D., Slot, R. E., Gouw, A. A., Teunissen, C. E., Scheltens, P., van der Flier, W. M. and Visser, P. J. (2017): Low normal cerebrospinal fluid Aβ42 levels predict clinical progression in nondemented subjects. Annals of neurology, 81(5), 749-753.

van Maurik, I. S., Slot, R., Verfaillie, S., Zwan, M. D., Bouwman, F. H., Prins, N. D., Teunissen, C. E., Scheltens, P., Barkhof, F., Wattjes, M. P., Molinuevo, J. L., Rami, L., Wolfsgruber, S., Peters, O., Jessen, F., Berkhof, J., van der Flier, W. M. and Alzheimer's Disease Neuroimaging Initiative (2019): Personalized risk for clinical progression in cognitively normal subjects-the ABIDE project. Alzheimer's research & therapy, 11(1), 33.

von Unger, H. (2014). Research ethics in qualitative research: principles, debates and open questions. In: Research ethics in qualitative research. Springer VS, Wiesbaden. S. 15-39.

Wolfsgruber, S., Polcher, A., Koppara, A., Kleineidam, L., Frölich, L., Peters, O., Hüll, M., Rüther, E., Wiltfang, J., Maier, W., Kornhuber, J., Lewczuk, P., Jessen, F. and Wagner, M. (2017): Cerebrospinal Fluid Biomarkers and Clinical Progression in Patients with Subjective Cognitive Decline and Mild Cognitive Impairment. Journal of Alzheimer's disease : JAD, 58(3), 939-950.

Bibliography Test battery:

MMSE

F. Folstein, S. E. Folstein, P. R. McHugh: Mini-Mental State (a practical method for grading the state of patients for the clinician). In: Journal of Psychiatric Research. 12, 1975.

Objective and Subjective Numeracy Scale

García-Retamero, R., & Galesic, M. (Eds.). (2013). Transparent communication of health risks: Overcoming cultural differences. New York: Springer.

Following: Making numbers matter: present and future research in risk communication.Fagerlin A, Ubel PA, Smith DM, Zikmund-Fisher BJ. Am J Health Behav. 2007

FLZM

Henrich, G. and Herschbach, P.: Questions on Life Satisfaction (FLZM) - A short questionnaire for assessing subjective quality of life. European Journal of Psycholo-gical Assessment, 2000

BRS

Smith, B. W., Dalen, J., Wiggins, K., Tooley, E., Christopher, P., & Bernard, J. (2008). The brief resilience scale: assessing the ability to bounce back. International journal of behavioral medicine, 15(3), 194-200.

BFI-10

Rammstedt, B., Kemper, C. J., Klein, M. C., Beierlein, C., & Kovaleva, A. (2014). Big Five Inventory (BFI-10).

HADS

Zigmond AS, Snaith RP. The hospital anxiety and depression scale. Acta Psychiatr Scand. 1983;67:361-70.

UCLA Loneliness

Hughes ME, Waite LJ, Hawkley LC, Cacioppo JT. A Short Scale for Measuring Loneliness in Large Surveys: Results From Two Population-Based Studies. Res Aging. 2004;26(6):655-672. doi: 10.1177/0164027504268574. PMID: 18504506; PMCID: PMC2394670.

SCD questionnaire

The SCD questionnaire designed to capture the SCD-plus criteria which are features of SCD that in the current state of knowledge are associated with increased likelihood of underlying AD pathology.

Following: Jessen F, Spottke A, Boecker H, et al. Design and first baseline data of the DZNE multicenter observational study on predementia Alzheimer's disease (DELCODE). Alzheimers Res Ther. 2018;10(1):15. Published 2018 Feb 7. doi:10.1186/s13195-017-0314-2

Jessen F, Amariglio RE, van Boxtel M, Breteler M, Ceccaldi M, Chételat G, et al. A conceptual framework for research on subjective cognitive decline in preclinical Alzheimer's disease. Alzheimers Dement. 2014;10:844-52.
